# Comprehensive epigenomic profiling reveals the extent of disease-specific chromatin states and informs target discovery in ankylosing spondylitis

**DOI:** 10.1016/j.xgen.2023.100306

**Published:** 2023-04-24

**Authors:** Andrew C. Brown, Carla J. Cohen, Olga Mielczarek, Gabriele Migliorini, Félicie Costantino, Alice Allcock, Connor Davidson, Katherine S. Elliott, Hai Fang, Alicia Lledó Lara, Alice C. Martin, Julie A. Osgood, Anna Sanniti, Giuseppe Scozzafava, Matteo Vecellio, Ping Zhang, Mary Helen Black, Shuwei Li, Dongnhu Truong, Julio Molineros, Trevor Howe, B. Paul Wordsworth, Paul Bowness, Julian C. Knight

**Affiliations:** 1 Wellcome Centre for Human Genetics, University of Oxford, Oxford OX3 7BN, UK; 2 MRC WIMM Centre for Computational Biology, MRC Weatherall Institute of Molecular Medicine, University of Oxford, John Radcliffe Hospital, Oxford OX3 9DS, UK; 3 Nuffield Department of Orthopaedics, Rheumatology, and Musculoskeletal Sciences, University of Oxford, Oxford OX3 7LD, UK; 4 Horizon Discovery (PerkinElmer) Cambridge Research Park, 8100 Beach Dr., Waterbeach, Cambridge CB25 9TL, UK; 5 Department of Biochemistry, University of Oxford, South Parks Road, Oxford OX1 3QU, UK; 6 UVSQ, INSERM UMR1173, Infection et Inflammation, Laboratory of Excellence INFLAMEX, Université Paris-Saclay, Paris, France; 7 Rheumatology Department, AP-HP, Ambroise Paré Hospital, Paris, France; 8 Shanghai Institute of Hematology, State Key Laboratory of Medical Genomics, National Research Centre for Translational Medicine at Shanghai, Ruijin Hospital affiliated with Shanghai Jiao Tong University School of Medicine, Shanghai 200025, China; 9 Centro Ricerche Fondazione Italiana Ricerca sull’Artrite (FIRA), Fondazione Pisana per la Scienza ONLUS, Via Ferruccio Giovannini 13, 56017 San Giuliano Terme (Pisa), Italy; 10 Chinese Academy of Medical Sciences Oxford Institute, Nuffield Department of Medicine, University of Oxford, Oxford OX3 7BN, UK; 11 Data Science, Population Analytics, Janssen R&D, Spring House, PA 19002, USA; 12 Data Science, External Innovation, Janssen R&D, London W1G 0BG, UK; 13 National Institute for Health Research, Comprehensive Biomedical Research Centre, Oxford OX4 2PG, UK

**Keywords:** ankylosing spondylitis, spondyloarthritis, epigenomics, functional genomics, transcriptomics, target discovery, chromatin interactions, gene regulation, genome-wide association study

## Abstract

Ankylosing spondylitis (AS) is a common, highly heritable inflammatory arthritis characterized by enthesitis of the spine and sacroiliac joints. Genome-wide association studies (GWASs) have revealed more than 100 genetic associations whose functional effects remain largely unresolved. Here, we present a comprehensive transcriptomic and epigenomic map of disease-relevant blood immune cell subsets from AS patients and healthy controls. We find that, while CD14^+^ monocytes and CD4^+^ and CD8^+^ T cells show disease-specific differences at the RNA level, epigenomic differences are only apparent upon multi-omics integration. The latter reveals enrichment at disease-associated loci in monocytes. We link putative functional SNPs to genes using high-resolution Capture-C at 10 loci, including *PTGER4* and *ETS1*, and show how disease-specific functional genomic data can be integrated with GWASs to enhance therapeutic target discovery. This study combines epigenetic and transcriptional analysis with GWASs to identify disease-relevant cell types and gene regulation of likely pathogenic relevance and prioritize drug targets.

## Highlights

• Comprehensive transcriptomic and epigenomic mapping in ankylosing spondylitis
• Disease-specific chromatin patterns seen upon integration of multiple modalities
• Evidence of functional SNPs via chromosome looping events at GWAS regions
• Gene and pathway prioritization facilitates future drug discovery

## Graphical abstract

**Figure:**
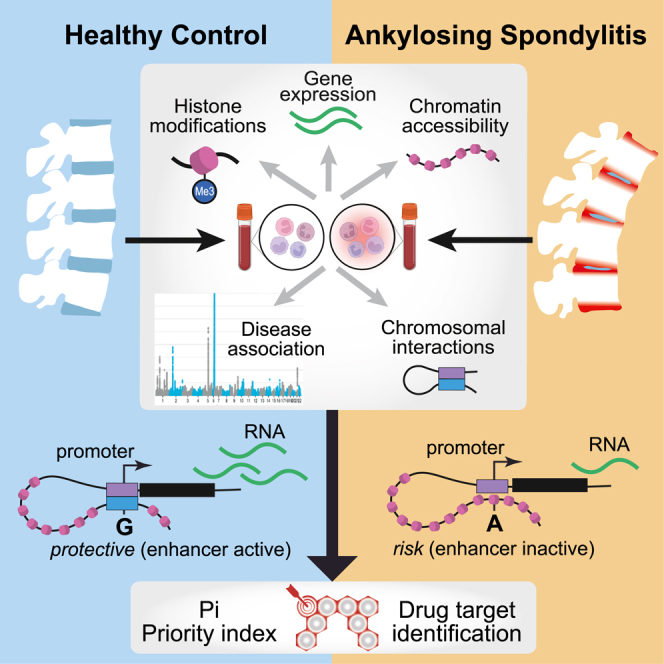

## Teaser

Brown et al. performed comprehensive transcriptomic and epigenomic mapping in ankylosing spondylitis. Comparison of blood immune cells from patients and healthy controls revealed disease-specific chromatin patterns with potential pathogenic effects via mechanisms such as chromosome looping. The authors used these results to prioritize genetic variants, genes, and pathways for drug discovery.

## Introduction

Ankylosing spondylitis (AS) is a common inflammatory arthritis characterized by inflammation of the sacroiliac joints and spinal entheses, which causes extensive new bone formation and vertebral fusion, resulting in pain, loss of movement, and disability.^1^^,^^2^ Combined with other systemic manifestations of the disease, such as inflammation of the gut, skin, and eyes, this leads to significant morbidity and disease burden.^3^^,^^4^ Twin and other family studies indicate that AS is highly heritable (**λ**s ∼50) with broad-sense heritability greater than 90%.^5^^,^^6^ This involves strong association with the major histocompatibility complex (MHC) allele HLA-B27^7^^,^^8^ and more than 100 other loci identified through genome-wide association studies (GWASs).^9^^,^^10^^,^^11^^,^^12^^,^^13^ Several of these associations implicate genes involved in interleukin-23 (IL-23)-driven inflammation and Th17 responses; these include *IL23R* (encoding the IL-23 receptor), *IL6R* (IL-6 receptor), *TYK2* (tyrosine kinase 2 receptor), and *IL27R*.^9^ In a few cases, such as *ERAP1* (endoplasmic reticulum aminopeptidase 1) and *IL23R*, functional non-synonymous single-nucleotide polymorphisms (SNPs) have been described.^9^^,^^13^^,^^14^ However, most AS associations involve non-coding SNPs, which may be regulatory in nature and act in a cell-specific manner to modulate a variety of epigenetic, transcriptional, and post-transcriptional mechanisms.^15^^,^^16^^,^^17^^,^^18^ Recently we demonstrated how AS-associated SNPs at *RUNX3* modulate the binding of transcription factors (TFs) and regulatory complexes in T cells and monocytes,^15^^,^^16^^,^^17^^,^^18^ but for other associated loci, the causal genes and pathways remain largely unresolved.^19^ The expression and co-ordination of regulatory mechanisms for genes involved in the disease pathophysiology are likely to be cell type specific. Here we focus on CD4^+^ and CD8^+^ T cells and monocytes from patients with active AS and healthy controls (HC) because these cell types have been implicated previously in the pathogenesis of AS,^20^^,^^21^^,^^22^^,^^23^^,^^24^^,^^25^^,^^26^ and previous studies have largely sampled whole peripheral blood mononuclear cells (PBMCs).^27^^,^^28^^,^^29^^,^^30^

The outlook for patients with more severe forms of AS has been greatly improved in recent years by the introduction of new biologic treatments inhibiting the inflammatory cytokines tumor necrosis factor alpha (TNF-α) and IL-17A. Nevertheless, fewer than half are likely to achieve sustained remission even with these targeted therapies,^31^ highlighting the need for patient stratification of potential responders and new therapeutic targets in AS. Human genetic evidence supporting the identification of therapeutic targets strongly increases the likelihood of success in late-stage clinical trials.^32^ We and others have shown that GWASs, in combination with functional genomic evidence and knowledge of network connectivity, can be used to prioritize target genes and pathways through, for example, the priority index (Pi) algorithm.^33^^,^^34^ Logistical and technical challenges have limited the number of studies generating omics data from patient samples to date, and it remains unresolved how best to maximize the value of such data through integration across assay modalities, including genetics.^35^

Here, we present a comprehensive map of the epigenomic landscape of AS defining the global transcriptome, chromatin accessibility, and enhancer- and promoter-associated histone modifications in disease-relevant subsets of immune cells from patients and HCs. We identify global changes in chromatin architecture in the AS disease state in monocytes and characterize specific GWAS loci to identify interactions between lead SNPs in enhancers and cognate genes, including prostaglandin E receptor 4 (*PTGER4*) and ETS proto-oncogene 1 (*ETS1*). Furthermore, we show how functional genomic evidence can be integrated with GWAS data through Pi to identify candidate therapeutic targets for future study.

### Design

#### Patient and control cohorts and experimental overview

**Figure 1:**
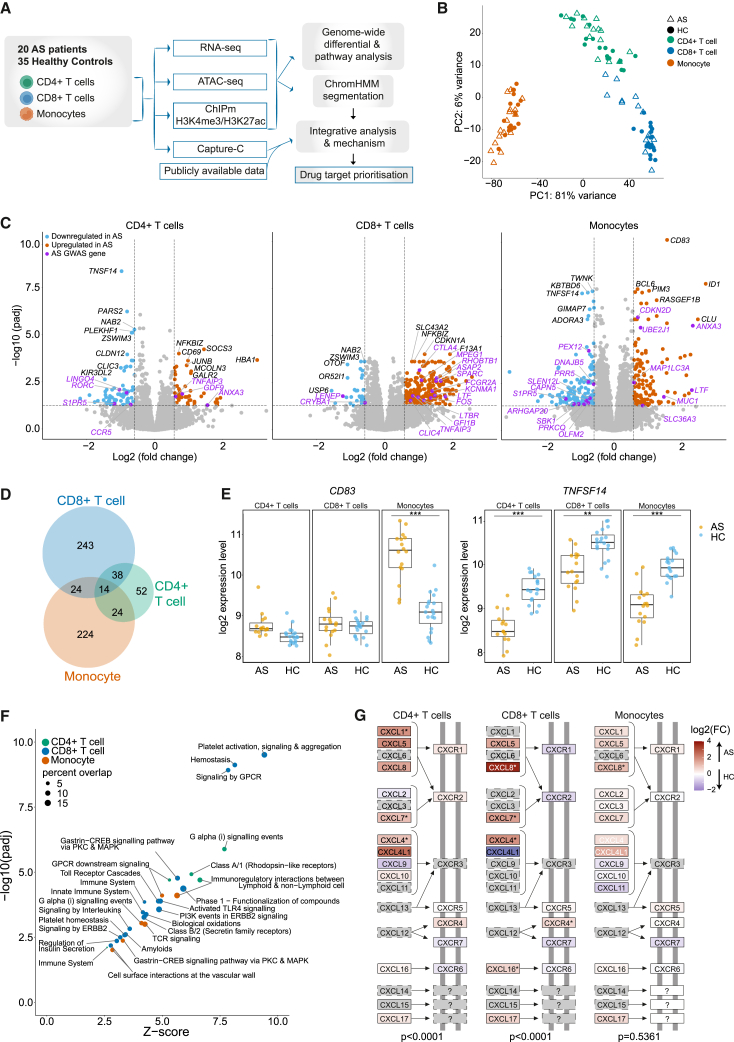
Gene expression levels consistently delineate cell type and show differences between AS patients and HCs (A) Workflow of the study. (B) PCA of RNA-seq data in CD4^+^ T cells, CD8^+^ T cells, and monocytes from AS patients and HCs. (C) Volcano plot showing differentially expressed genes calculated using DEseq2 (padj < 0.05, FC > 1.5) between AS patients and HCs in CD4^+^ T cells, CD8^+^ T cells, and monocytes. Genes in AS-associated GWAS regions are purple. Red genes are upregulated and blue downregulated in AS patients. CD4^+^ T cells had 122 differentially expressed genes, CD8^+^ T cells 299 genes, and monocytes 300 genes. (D) Cell type specificity of differentially expressed genes; numbers of differentially expressed genes are given. (E) Examples of differential gene expression at *CD83* and *TNFSF14*; ∗∗padj < 0.01, ∗∗∗padj < 10^−7^ (from DEseq2). (F) Enriched pathways in the Reactome database (FDR < 0.01 from XGR output) from significant differentially expressed genes in each cell type. Dot size represents percentages of genes represented in that pathway, and colors represent cell types. (G) CXC subfamily of the Kyoto Encyclopedia of Genes and Genomes (KEGG) “cytokine-cytokine reception interaction” pathway colored by gene expression log_2_ FC. Significantly differentially expressed genes are marked by asterisks. The p value of CXC family subset over-representation is shown below for each cell type, calculated by chi-squared test with Yates’ correction. See also Figure S1 and Table S3.

To generate a comprehensive functional genomic and epigenomic atlas of the immune response in peripheral blood of AS patients, we recruited 20 adult patients with active disease who were naive to biologic therapy and fulfilled diagnostic criteria for AS and 35 HCs recruited locally or from the Oxford Biobank (Table S1; Table S2; STAR Methods). Cell populations of interest (CD4^+^ and CD8^+^ T cells and CD14^+^ monocytes) were freshly isolated from peripheral blood by positive selection using immunomagnetic cell separation with more than 98% purity (Figure S1; STAR Methods). Each cell type was then processed immediately for total RNA sequencing (RNA-seq), chromatin accessibility (assay for transposase-accessible chromatin with next-generation sequencing [ATAC-seq]), informative histone modifications for promoter (histone H3 lysine 4 trimethylation [H3K4me3]) and enhancer (histone H3 lysine 4 trimethylation [H3K27ac]) activity (ChIPmentation), and high-resolution chromosomal conformation capture (Capture-C) (Figure 1A; Table S2; STAR Methods).

## Results

### Differential gene expression in active AS is cell type specific

We first investigated the nature of differential gene expression between AS patients and HCs for specific immune cell types. We focused on three major immune cell types previously implicated in AS (CD4^+^ and CD8^+^ T cells and CD14^+^ monocytes).^20^^,^^21^^,^^22^^,^^23^^,^^24^^,^^25^^,^^26^ Analysis of gene expression by RNA-seq comparing the three cell types for each individual showed that gene expression segregated by cell type more strongly than by disease state (Figures 1B and S1B). However, for each cell type, we found hundreds of differentially expressed genes between AS patients and HC (CD4^+^ T cells, 122 genes; CD8^+^ T cells, 299 genes; monocytes, 300 genes; padj (adjusted p value) < 0.05, fold change [FC] > 1.5) (STAR Methods; Figures 1C and S1C; Table S3). The majority of differentially expressed genes were cell type specific (Figure 1D), and where differentially expressed genes involved more than one cell type, the direction of effect was the same in the majority of cases. For example, *CD83* (encoding CD83, a cell-surface glycoprotein involved in regulation of antigen presentation) has significantly higher expression only in AS patient monocytes, *TNFSF14* (encoding TNF superfamily member 14) has significantly lower expression in AS patients in all three cell types (Figure 1E), and *SCAMP5* is an example of a gene upregulated in CD8^+^ T cells and downregulated in CD4^+^ T cells. We defined disease-enriched pathways from differentially expressed genes using eXploring Genomic Relations (XGR)^36^ (STAR Methods). All cell types showed enrichment of immune-related pathways and G protein-coupled receptor (GPCR) signaling pathways in AS (Figure 1F). We identified significant upregulation of the CXC subfamily of chemokine receptors in CD4^+^ and CD8^+^ T cells (Figures 1G and S1D), which links to the important role of IL-17-producing cells in AS pathogenesis. We investigated cell subset composition by deconvolution of RNA-seq data using CIBERSORTx^37^ and found no difference in abundance of the three cell types or major cell subsets within these when comparing AS patients and HCs (Figure S1E).

### Cell-type-specific epigenomic marks show limited differences between AS patients and controls

**Figure 2:**
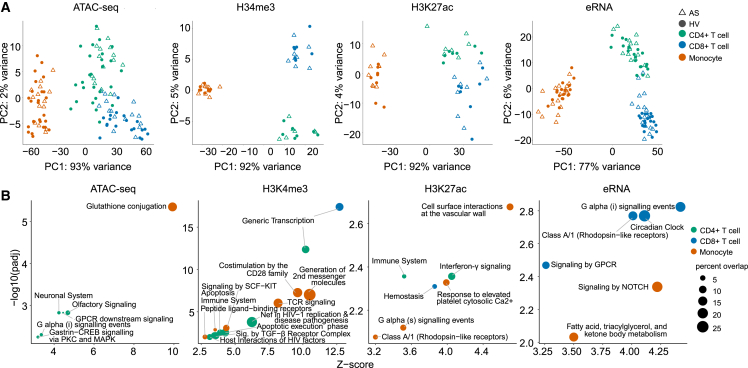
Individual epigenomic mapping methods in immune cell subsets have limited capacity to differentiate AS patients and HCs (A) PCA of genome-wide distribution of ATAC-seq, ChIPm H3K4me3, ChIPm H3K27ac, and eRNA peaks in CD4^+^ T cells, CD8^+^ T cells, and monocytes from AS patients and HCs. (B) Enriched Reactome pathways (FDR < 0.01 from XGR output) within genes associated with the top 200 differentially expressed peaks in each cell type. Note that each modality is plotted with different x and y scales to maximize clarity. Dot size represents percentages of genes represented in that pathway, and colors represent cell types. Numbers of significant differential peaks (padj < 0.05, FC > 1.5) are shown at the top left for each modality, colored by cell type. See also Figure S2 and Table S4. Differential ATAC-seq peaks between AS patients and HCs in CD4+ T cells, CD8+ T cells, and monocytes, related to Figure 2, Table S5. Differential ChIP-seq peaks between AS patients and HCs in CD4+ T cells, CD8+ T cells, and monocytes, related to Figure 2, Table S6. Differentially expressed eRNAs between AS patients and HCs in CD4+ T cells, CD8+ T cells, and monocytes, related to Figure 2, Table S7. Differential ChromHMM regions between AS patients and HCs in monocytes, related to Figure 3.

We then resolved genomic regulatory features in CD4^+^ T cells, CD8^+^ T cells, and monocytes from AS patients. To do this, we assayed open chromatin with ATAC-seq and enrichment of H3K4me3 and H3K27ac histone modifications using ChIPmentation (ChIPm) and identified non-coding enhancer RNAs (eRNAs) within ATAC peaks outside of coding genes (STAR Methods; Figures S2A and S2B). Each omics modality showed cell type specificity on principal-component analysis (PCA) that outweighed the effect of disease state (Figure 2A). Only a very small number of significant differential signals were observed between AS patients and HCs (Figure 2B; Table S4. Differential ATAC-seq peaks between AS patients and HCs in CD4+ T cells, CD8+ T cells, and monocytes, related to Figure 2, Table S5. Differential ChIP-seq peaks between AS patients and HCs in CD4+ T cells, CD8+ T cells, and monocytes, related to Figure 2, Table S6. Differentially expressed eRNAs between AS patients and HCs in CD4+ T cells, CD8+ T cells, and monocytes, related to Figure 2), and disease state could not be clearly distinguished on PCA (Figure S2C). The transcription start site (TSS) score for ATAC and chromatin immunoprecipitation (ChIP) correlated with expression levels of their corresponding gene (Figure S2D). We assigned differential ATAC, ChIPm, and eRNA signals to genes by proximity or overlap with promoter capture Hi-C (PCHi-C) looping interactions identified in relevant cell types^38^ (STAR Methods). Pathway enrichment analysis of genes linked to the top 200 differential regions between AS patients and HCs for each modality implicated immunological pathways and GPCR signaling along with transcriptional pathways and NOTCH signaling across cell types and modalities, consistent with our analysis of differentially expressed genes (Figure 2B).

### Disease-specific regulatory chromatin states are found in monocytes from patients with AS

**Figure 3:**
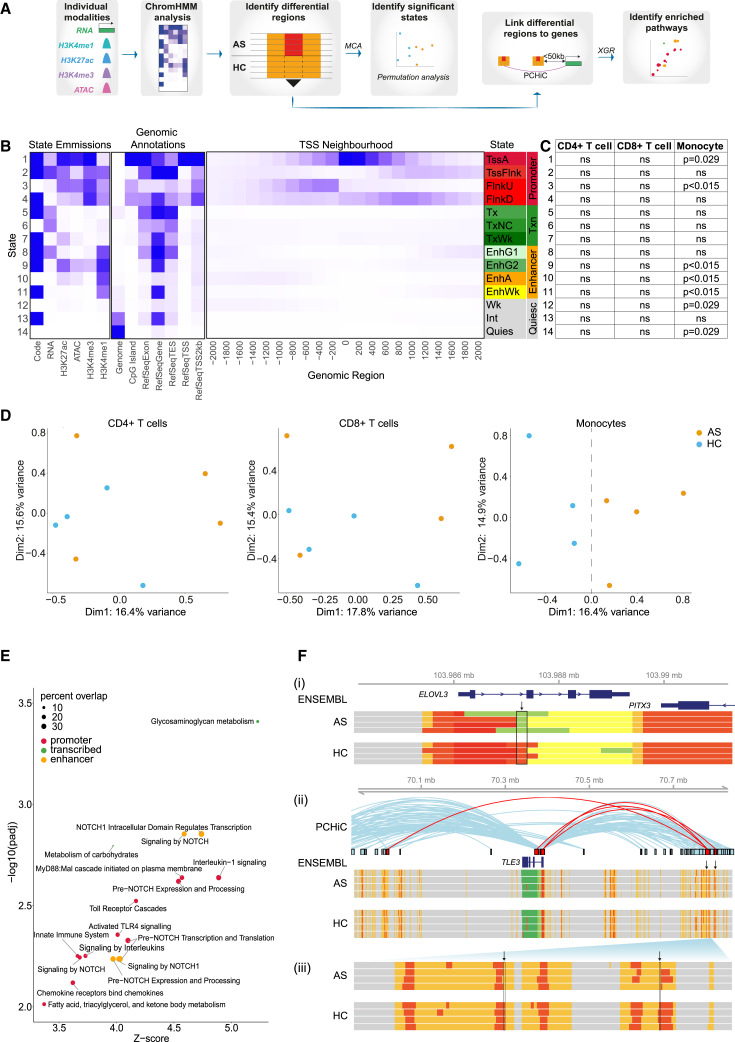
Disease-specific regulatory regions are found in monocytes and implicate NOTCH signaling in AS pathogenesis (A) Workflow of the ChromHMM analysis. (B) ChromHMM emission model showing 14 states annotated according to state emissions, genomic annotations, and TSS neighborhood. State names were assigned according to predicted function, aligned to The Encyclopedia of DNA Elements (ENCODE) labels where possible. Promoter states: TssA, active TSS; TssFlnk, flanking promoter; FlnkU, upstream of TSS; FlnkD, downstream of TSS. Transcribed (Txn) states: Tx, transcription; TxNC, non-coding transcription; TxWk, weak transcription. Enhancer states: EnhG1, strongly transcribed, weak enhancer; EnhG2, weakly transcribed, strong enhancer; EnhA, enhancer; EnhWk, weak intronic enhancer. Quiescent (Quiesc) states: Wk, low/weak enhancer; Int, introns; Quies, quiescent. (C) Significance of disease-specific ChromHMM segment frequency for each state in each cell type, calculated using permutation analysis (ns, not significant). Only monocytes contained significantly over-represented states. (D) MCA showing overall distribution of ChromHMM states within each cell type comparing AS patients and HCs. The monocyte plot dotted line at x = 0 delineates AS and HC samples. (E) Enriched pathways in the Reactome database (FDR < 0.01, XGR output) performed on genes linked to promoter-like states, transcribed regions, or enhancer-like states in monocytes. Dot size represents percentage of genes represented in that pathway. (F) Visualization of differential ChromHMM regions (vertical arrows) at (i) the promoter of *ELOVL3* and (ii) enhancer of *TLE3*, with (iii) magnified region of the *TLE3* differential enhancer. ChromHMM states in AS patients and HCs are colored as in (B). PCHiC looping interactions^38^ are shown, with loops intersecting differential ChromHMM segments in red. See also Figure S3 and Table S8.

We further investigated whether there were disease-specific differences in the chromatin landscape by maximizing the informativeness of different sources of omics information using ChromHMM^39^ (Figure 3A), a machine learning algorithm that enables definition of chromatin conformational states based on combinations of regulatory features. The derived emission model consisted of 14 chromatin states (Figure 3B) in four functional categories: promoter (states 1–4), transcribed (states 5–7), enhancer (states 8–11), and quiescent (states 12–14). Multiple correspondence analysis (MCA) on global ChromHMM data revealed major differences between cell types, albeit with considerable variation between individuals (Figure S3A). Within each cell type, we identified thousands of 200-bp segments that were assigned to different chromatin states in AS patients and HCs (Table S7). We found that differential segments in two promoter states (1 and 3), three enhancer states (9, 10, and 11), and two quiescent states (12 and 14) were significantly over-represented in AS monocytes (Figure 3C), as determined by permutation analysis (Figure S3B). Consistent with this, MCA comparison of AS patients and HCs showed separation on dimension (dim) 1 in monocytes but not CD4^+^ and CD8^+^ T cells (Figure 3D). Within monocytes, all states except state 14 (quiescent) showed separation of AS patients and HCs on dim 1 or dim 2 (Figure S3C). In contrast, only three states showed separation in CD8^+^ T cells (states 7, 8, and 11), and none in CD4^+^ T cells. Comparative state transitions between patients and HCs revealed a complex pattern of differential states, including enrichment of enhancer state 10 (EnhA) in AS patients corresponding to promoter state 1 (TssA) in HCs (Figure S3D). Overall, this analysis demonstrates significant changes in the epigenomic landscape of active AS disease, notably in monocytes.

We next sought to determine which molecular pathways might be altered by these global changes in monocyte chromatin architecture. We assigned the differential ChromHMM fragments to genes based on proximity or PCHi-C looping events^38^ (STAR Methods; Table S7) and performed pathway enrichment analysis (Figure 3E). The highest number of enriched pathways contained genes linked to promoter state, followed by those linked to enhancer state. Only two pathways were enriched in genes linked to transcribed states and none with quiescent states. Six of 15 enriched pathways from promoter and enhancer states related to NOTCH signaling (Figure 3E), further implicating this pathway in monocytes in AS. To aid interpretation of these findings, we further defined by flow cytometry which monocyte subpopulations were represented in our sorted monocyte population, showing that these are 80% CD14^+^ CD16^+^ classical monocytes with the remainder intermediate and non-classical monocytes (Figure S3E).

To illustrate our results, we show two examples where differential ChromHMM segments correlate with alterations in gene expression (Figure 3F). *ELOVL3* (encoding elongation of very-long-chain fatty acids) is involved in fatty acid metabolism and downregulated in psoriasis,^40^ a common extra-articular manifestation of AS.^41^ The *ELOVL3* promoter is marked by state 1 (TssA, promoter-like) in AS patients and HCs, but this mark spans a shorter genomic interval in AS patients (Figure 3F). Consistent with this, expression of *ELOVL3* is lower in AS patients (padj = 0.0003). *TLE3* (encoding transducin-like enhancer family member 3) is a transcriptional co-repressor involved in the NOTCH signaling pathway. We identified up- and downstream enhancers that form looping interactions with the *TLE3* promoter (Figure 3F). The enhancers are defined by ChromHMM state 10 (enhancer) containing punctate regions of state 1 (promoter) that correspond with non-coding eRNA transcription. In two segments these promoter elements are narrower in AS patients compared with HC, which may contribute to the observed reduction in *TLE3* expression in AS patients (padj = 0.045).

### Regulatory chromatin signatures are enriched at AS GWAS regions

**Figure 4:**
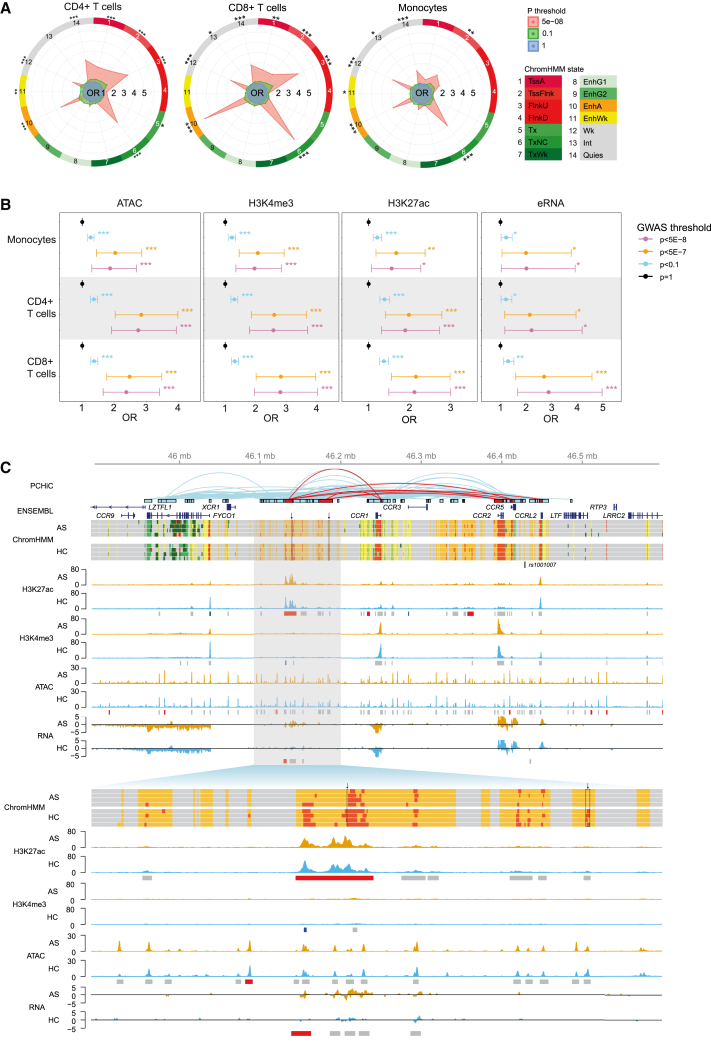
Differential chromatin regions are enriched at GWAS loci (A) Enrichment of differential ChromHMM differential regions at AS-associated GWAS loci^10^ with association p value thresholds as indicated in CD4^+^ T cells, CD8^+^ T cells, and monocytes. ∗∗∗p < 0.001, ∗∗p < 0.01, ∗p < 0.05. OR, odds ratio. (B) Enrichment of ATAC, H3K4me3, H3K27ac, and eRNA peaks at AS-associated GWAS loci^9^ in CD4^+^ T cells, CD8^+^ T cells, and monocytes; GWAS association p value thresholds are indicated. Error bars represent 95% confidence interval of the OR (from GARFIELD^42^). ∗∗∗p < 0.001, ∗∗p < 0.01, ∗p < 0.05. (C) Visualization of multiple epigenomic datasets at chr3p21 (top, chr3:45890000-46600000; bottom, chr3:46090000-46200000). PCHiC looping events^38^ that intersect regions of differential chromatin are indicated in red. Selected gene transcripts from the Ensembl database are shown. ChromHMM is shown for 4 AS patients and 4 HCs with colors as in Figure 3B. The lead AS-associated SNP *rs1001007*^10^ is shown. Representative ATAC, H3K4me3, and H3K27ac tracks (RPKM) and total RNA tracks (log2 count) are shown for AS patients (orange) and HCs (blue), with called peaks marked by gray boxes and differential peaks shown in red (upregulated in AS) or blue (downregulated in AS). See also Figure S4.

Having generated comprehensive epigenomic maps in three cell types from AS patients and HCs, we next addressed whether this could inform the functional basis of observed genetic associations in AS from GWASs, specifically seeking evidence to implicate/delineate the gene(s) responsible for the genetic association. We identified 35 differentially expressed genes located within GWAS regions (<500 kb from the lead SNP), of which 31 were cell type specific (Figure 1C; STAR Methods). We found that regulatory ChromHMM states are over-represented in or near AS GWAS regions, and this is independent of the HLA-B27 association (Figures 4A and S4). Enhancer state 10 is enriched near GWAS regions in all three cell types, while in monocytes, states 2, 6, 10, 11, 12, 13, and 14 are all enriched in GWAS regions. We also found significant enrichment of ATAC, H3K4me3, and H3K27ac signals and eRNAs at GWAS loci in all cell types (Figure 4B).

The chr3p21.31 locus illustrates the intersection between an AS genetic association (*rs1001007*^10^) and disease-specific chromatin state in monocytes (Figure 4C). This intergenic SNP is located between *CCR5* and *CCRL2*, but this locus has not been fine mapped to establish a functional variant and modulated gene in AS. Consequently, we interrogated the whole monocyte-specific topologically associating domain (TAD) and discovered several regions with disease-specific chromatin signatures. We found evidence of an intergenic region between *XCR1* and *CCR1* that has an enhancer-like profile (ChromHMM state 10, EnhA) in AS patients with a promoter-like profile (ChromHMM state 3, FlnkU) in HCs, consistent with observed differences in eRNA and H3K27ac between cases and controls. Analysis of chromatin interaction data shows that this region is involved in DNA looping to *CCR1*, *CCR2*, *CCR5*, and *CCRL2* but not with *CCR3*. Looping events correlate with expression of the cognate genes in monocytes. These findings indicate complex regulation of the CCR gene cluster in monocytes with epigenetic changes specific to AS.

### Chromosome looping interactions from AS-associated SNPs in enhancers implicate disease-relevant genes

**Figure 5:**
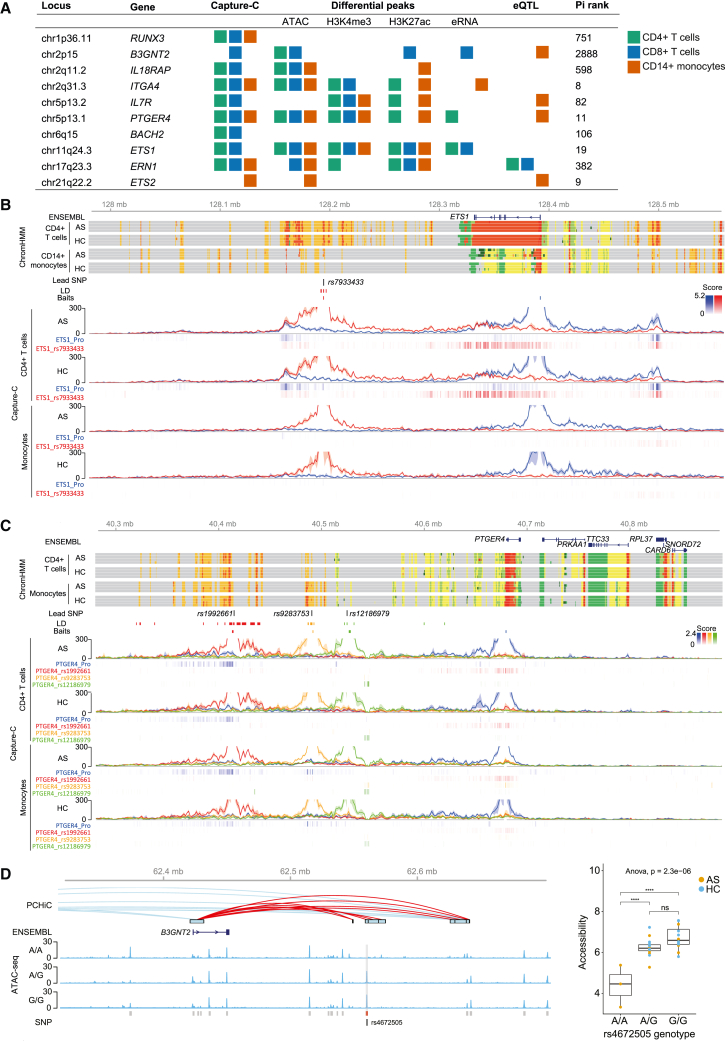
Chromosome looping interactions link genes with genetically associated SNPs at enhancers (A) Summary of epigenomic evidence at 10 GWAS loci where enhancer-gene interactions are observed. Colored squares indicate the presence of Capture-C interactions, differential ATAC, H3K4me3, H3K27ac, eRNA peaks, or eQTLs^45^ in each cell type. Pi rank shows priority ranking among 17,000 genes (Figure 6). (B) Visualization of the *ETS1* locus (chr11:127980000–128560000). (C) Visualization of the *PTGER4* locus (chr5:40280000–40890000). The following data are shown for CD4^+^ T cells and monocytes. Ensembl: selected transcripts of Ensembl genes. ChromHMM: ChromHMM data from four AS patients and four HC are shown with colors as in Figure 3B. Lead SNP: Position of lead GWAS SNPs from International Genetics of Ankylosing Spondylitis Consortium (IGAS) et al.^9^ and/or Ellinghaus et al.^10^ as per Table 1. LD: SNPs in LD (r^2^ > 0.8) with lead SNP. Baits: position of Capture-C baits (see also Table S9). Capture-C: mean interaction count (n = 3) and 1 SD shading, with PeakY scores colored according to bait for AS patients and HCs in CD4^+^ T cells, CD8^+^ T cells, and monocytes. (D) Genetic association with chromatin openness at *B3GNT2.* Left: visualization of the B3GNT2 locus (chr2:62320000–62700000) in CD8^+^ T cells. PCHiC: PCHiC looping events.^38^ ENSEMBL: B3GNT2 gene. ATAC-seq: representative ATAC tracks for AS patients with each rs4672505 genotype. Called peaks are shown in gray with the differential peak chr2:62559366–62561099 in red. SNP: position of *rs4672505.* Right: association of ATAC-seq peak chr2:62559366–62561099 limma-corrected counts with the *rs4672505* genotype calculated by ANOVA with Tukey post-test*.* ∗∗∗∗p < 0.0001. See also Figures S5–S14 and Table S9.

To further substantiate the relationship between disease-associated SNPs and likely modulated genes, we mapped chromosome looping events at GWAS loci in patient samples at high resolution (compared with PCHi-C). We performed Capture-C on CD4^+^ T cells, CD8^+^ T cells, and monocytes from AS patients and controls (STAR Methods). Baits were designed at promoters of genes with known immune roles within AS GWAS regions. We found that 18 of 44 promoter viewpoints assayed demonstrated chromatin interactions in at least one cell type, although no differences were found between AS patients and HCs. Follow-up Capture-C experiments were performed with baits at AS-associated SNPs to demonstrate reciprocal interactions between SNPs and promoters. Overall, nine reciprocal interactions were found between promoters and regions containing GWAS SNPs, nine of which were marked as enhancers (state 10 or 11) in our ChromHMM analysis in the same cell type (Table 1; Figures 5 and S5–S14). These loci also contained differential ATAC, H3K4me3, H3K27ac, or eRNA peaks, and five overlapped expression quantitative trait loci (eQTLs) in the same cell types^43^ (Figure 5A).

**Table 1:** Evidence of enhancer-gene interactions at GWAS loci

Locus	Lead SNPs	Interacting gene	Gene function	Gene-SNP distance (bp)	Other evidence	Putative mechanism	Figure
chr1p36.11	*rs6600247*^a^^,^^b^	*RUNX3* (RUNX family transcription factor 3)	TF in T cell differentiation	13,612	functional evidence of disrupted TF binding^15^^,^^16^^,^^17^^,^^18^	SNPs alter TF binding; local looping interactions	Figure S5
chr2q11.2	*rs4851529*^a^^,^^b^	*IL18RAP* (IL-18 receptor accessory protein)	component of IL-18 receptor, binds pro-inflammatory cytokine	387,952	–	long-range enhancer	Figure S6
chr2q31.3	*rs12615545*^a^	*ITGA4* (integrin subunit alpha 4)	integrin component, role in cell motility and migration	273,165	long non-coding RNA in CD4/CD8	long-range enhancer	Figure S7
chr5p13.2	*rs11742270*^a^	*IL7R* (IL-7 receptor)	component of IL-7 receptor, binds pro-inflammatory cytokine	1,738	regulatory and splice variant SNPs^20^ eRNA present	two signals: regulatory SNP; splicing SNP controls soluble IL-7R production	Figure S8
chr5p13.1	*rs12186979*,^a^*rs1992661*^b^	*PTGER4* (prostaglandin E receptor 4)	prostaglandin receptor, role in IL-23 and TNF pathways	155,171	functional SNP^44^; eRNAs present; differential ChromHMM at promoter	long-range enhancer	Figures 5 and S9
chr6q15	*rs17765610*^a^	*BACH2* (BTB domain And CNC homolog 2)	TF, T cell regulation	0	–	long-range enhancer within gene	Figure S10
chr11q24.3	*rs7933433*^b^	*ETS1* (ETS proto-oncogene 1)	TF, regulates cytokines and chemokines	134,204	eRNA present	long-range enhancer	Figures 5 and S11
chr17q23.3	*rs196941*^b^	*ERN1* (endoplasmic reticulum to nucleus signaling 1)	unfolded protein response	0	–	intronic enhancer	Figure S12
chr21q22.2	*rs2836883*,^a^*rs9977672*^b^	*ETS2* (ETS proto-oncogene 2)	TF, T and B cell regulation	269,866	eRNA present	long-range enhancer	Figure S13

All SNPs except *rs1992661* were used as baits in the Capture-C experiment. See also Figures 5 and S5–S14.

a Lead SNP for each locus from Cortes et al.^9^

b Lead SNP for each locus from Ellinghaus et al.^10^

Two loci illustrate SNP-promoter interactions. *ETS1* encodes ETS proto-oncogene 1, a TF with numerous roles in immune cells, including regulation of cytokine and chemokine gene expression.^46^ Our ChromHMM analysis showed enhancer regions flanking the *ETS1* gene, and Capture-C analysis showed looping events between the enhancer overlapping the AS-associated lead SNP *rs7933433*^10^ and *ETS1* promoter in CD4^+^ and CD8^+^ T cells but not monocytes (Figures 5B and S11). *PTGER4* encodes prostaglandin receptor E4, a GPCR whose expression is associated with disease severity in AS.^47^ We observed an interaction between the promoter of *PTGER4* and the enhancer overlapping rs1992661 (AS GWAS lead SNP^10^) in all cell types (Figures 5C and S9). We detected an interaction between an enhancer encompassing the known functional SNP *rs9283753*^48^ and the *PTGER4* promoter specifically in monocytes and only with the promoter bait (Figures 5C and S9). There was no detectable interaction between the lead SNP *rs12186979* and *PTGER4* gene in any of the cell types*.* These results support a model where associated SNPs lying in an enhancer region interact with a distal gene promoter via a chromatin looping event. This, combined with additional local disease-context-specific epigenomic modifications, may lead to alterations in cognate gene expression.

We were interested to explore whether the genotypes of individual SNPs were associated with alterations in chromatin structure. The study was underpowered to perform such an analysis genome wide, but we were able to analyze the effect of individual lead AS-associated SNPs. In doing so, we identified an association between rs4672505 and ATAC-seq peak chr2:62559366–62561099, where the A risk allele correlates with a reduced ATAC-seq signal in CD8^+^ T cells (Figure 5D). Analysis of next-generation sequencing (NGS) reads from the 16 heterozygous individuals with at least 5 mapping reads showed that 99.4% of ATAC peak reads encoded the G allele, strongly suggesting that the risk A allele prevents chromatin opening. This finding was not driven by mapping bias because A was the reference allele in the hg19 build used in this analysis. This SNP lies at chr2p15 and overlaps a putative enhancer that forms a looping interaction with B3GNT2 identified by publicly available PCHi-C (Figure 5D). rs4672505 is associated with AS, Crohn disease, and psoriasis^10^ and is an eQTL for *B3GNT2*.^45^

### Use of disease-specific functional genomic datasets enhances drug target discovery in AS

**Figure 6:**
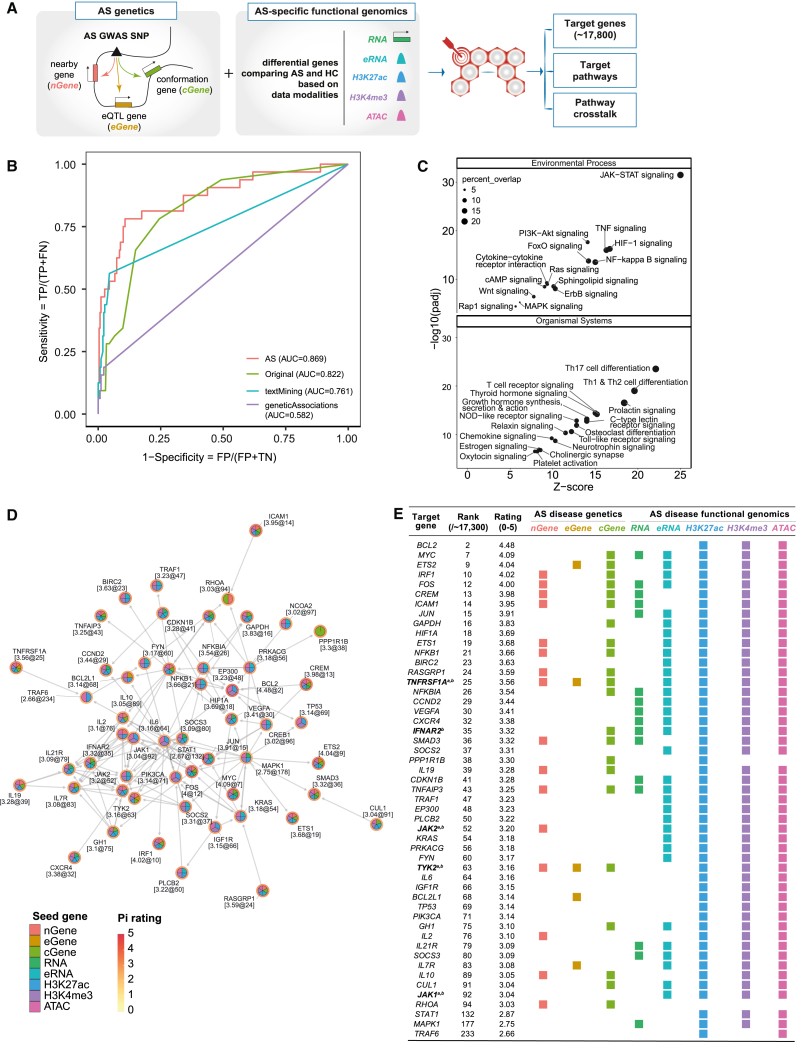
Prioritization of new drug targets in AS (A) Overview of the modified Pi prioritization algorithm. (B) Inclusion of disease-specific datasets (AS) improves the Pi algorithm output relative to the original Pi algorithm (Original) and outperformance relative to Open Targets textMining and geneticAssociations. AUC, area under the curve. (C) Prioritized target pathways (FDR < 0.05) based on KEGG pathway enrichment analysis using the top 1% prioritized genes. Dot size represents percentages of genes represented in that pathway. (D) Identification of pathway crosstalk; that is, a network of highly prioritized and interconnecting genes defined by KEGG interaction data, with nodes segmented by predictor/evidence types. (E) Table summarizing the prioritization and evidence information associated with pathway crosstalk genes. Colored boxes underneath each predictor type represent the datasets in support. Existing therapeutic targets are indicated for ^a^AS and ^b^other autoimmune conditions. See also Figure S15.

The final aim of our study was to prioritize new therapeutic targets in AS. We previously developed Pi, a genetics-led approach that annotates GWASs with functional genomic data to prioritize therapeutic targets across a range of immune-mediated diseases.^33^^,^^34^ We modified the underlying algorithm of Pi to include our new AS-specific functional genomic datasets (Figure 6A; STAR Methods), the algorithm previously having been limited to non-disease-context functional genomics data, and assessed whether this inclusion increased the power to identify potential therapeutic targets. In the original algorithm, we used disease-specific genetic associations to define seed (core) genes, including (1) nearby genes (nGene) using genomic proximity and organization, (2) expression-associated genes (eGene) integrating eQTL datasets, and (3) conformation genes (cGene) using PCHi-C datasets. Here, we added five types of AS-specific functional genomic predictors using data from this study (denoted RNA, eRNA, H3K27ac, H3K4me3, and ATAC). The AS-specific expression predictor (RNA) was generated based on differential gene expression, and AS-specific epigenomic predictors (ATAC, H3K4me3, H3K27ac, and eRNA) were prepared on the basis of differential peaks linked to genes as above.

We benchmarked the performance of the Pi algorithm with and without the AS-specific functional genomic predictors to prioritize currently approved drug targets for AS versus simulated negative targets (STAR Methods). This showed that inclusion of disease-specific data improved the predictive power (area under the curve [AUC] = 0.869) compared with the original prediction (AUC = 0.822) and the state-of-the-art approach (Open Targets,^49^ including text mining [AUC = 0.761] and genetic associations [AUC = 0.582] from the Open Targets Genetics Portal^50^) (Figure 6B). As expected, combined use of predictors performed much better than each predictor alone (Figures 6B and S15A). Among the top 1% of prioritized genes (of >17,000 ranked genes) were the known AS drug targets *IL23R*, *JAK2*, and *TYK2* (Figure S15B). Pathway enrichment analysis of the top 1% of genes identified pathogenic AS pathways, such as Janus kinase-signal transducer and activator of transcription (JAK-STAT) signaling (false discovery rate [FDR] = 3.3 × 10^−32^), TNF signaling (FDR = 1 × 10^−16^) and Th17 cell differentiation (FDR = 3.1 × 10^−24^) (Figure 6C). The significantly enriched pathways also included cytokine-cytokine receptor interaction, Toll-like receptor signaling, ErbB signaling, chemokine signaling, and T cell receptor (TCR) signaling, which were consistent with our findings from the RNA-seq data (Figure S2C). Pathway crosstalk analysis for potential therapeutic intervention identified a network of 53 interconnecting genes (Figure 6D), including genes that are already therapeutic targets in AS and other autoimmune conditions, such as *JAK1* and *TYK2*, alongside yet unexplored genes. The list of genes within this network (Figure 6E) are highly prioritized as candidates for therapeutic intervention, providing an input for the drug development pipelines and further study.^51^

## Discussion

### Summary of findings

We performed transcriptomic and epigenomic profiling in specific primary immune cell populations isolated from carefully phenotyped patients with active AS. This revealed disease-specific changes in the chromatin landscape and differential regulatory signatures significantly enriched near AS-associated loci. Pathway analysis indicated the importance of NOTCH and chemokine receptor signaling in AS in addition to known disease-associated pathways. Capture-C identified physical interactions between associated SNPs lying in enhancer regions and nearby gene promoters. Taken together, these results provide a map of the epigenetic landscape in AS and evidence of the mechanisms by which genetic associations can alter immune cell function in AS. Earlier studies have compared gene expression between AS patients and HCs in PBMCs.^27^^,^^28^^,^^29^^,^^30^ By looking at individual cell types, in this study we demonstrated a key role of monocytes in the pathogenesis of AS. This work highlights the importance of looking for functional effects of SNPs in the appropriate cellular and disease contexts in AS and other immune-mediated conditions.

### Identification of regulatory disease-associated SNPs and cognate genes

When analyzing the effects of a genetic variant with putative enhancer-modifying activity, a key component is to understand which gene(s) are regulated by that enhancer. Here, we described 10 enhancers that overlap AS-associated SNPs and that interact with a gene promoter within the same TAD (Table 1; Figures 5 and S5–S14). These enhancers share common features such as eRNA expression, activating histone marks, and open chromatin marks.

We discovered enhancer-gene interactions with two members of the ETS proto-oncogene (ETS) family of TFs, *ETS1* and *ETS2* (Figures 5, S11, and S13). At the *ETS1* locus, we observed chromatin looping interactions with dual enhancers, up- and downstream of the gene, specifically in T cells, of which the downstream enhancer overlaps an AS-associated lead SNP (*rs7933433*^10^). A monocyte-specific interaction was observed between an enhancer and the *ETS2* promoter, which overlaps the 99% credible set comprising 5 AS-associated SNPs^52^ that are also eQTLs in monocytes.^43^ *ETS1* and *ETS2* enhancers exhibit non-coding eRNA transcription. *ETS1* and *ETS2* are TFs expressed across various immune cell types and have roles including regulation of T cell subset differentiation.^53^ ETS1 regulates the expression of IL-7R^54^ (encoding the IL-7Rα subunit), which is also associated with AS,^20^ and *RUNX3*, which encodes Runt-related TF 3, a TF involved in T cell function with known AS-associated functional variants.^15^^,^^16^^,^^17^^,^^18^ *ETS1*, *ETS2*, and *IL7R* are in the Pi network output (Figure 6D), indicating that they form part of an important functional pathway with strong possibility for therapeutic intervention.

Gene-SNP interactions were also observed at the *PTGER4* locus (Figures 5 and S9). *PTGER4* is widely expressed throughout the immune system and is involved in the IL-23 and TNF-α pathways.^55^ It is also expressed in osteoclasts and could potentially have a role in new bone formation in AS.^56^ GWASs have found two independent associations at the *PTGER4* locus.^9^ Tewhey et al.^44^ described a functional SNP (*rs9283753*) that lies in an enhancer and alters *PTGER4* expression in lymphoblastoid cell lines. Our data show a chromatin looping interaction between AS-associated SNP rs1992661 and the *PTGER4* promoter in all three cell types and a T cell-specific interaction between rs9283753 and the *PTGER4* promoter. Our ChromHMM analysis shows that these SNPs overlap enhancer marks in the same cell types. Taken together, these data strongly support a functional role of rs1992661 and rs9283753 in regulation of *PTGER4*.

We found allele-specific differences in the ATAC-seq signal for an AS GWAS-associated SNP, rs4672505. This variant has been associated previously with differential abundance of *B3GNT2*, a poly-N-acetyllactosamine synthase in whole blood.^45^ B3GNT2 is upregulated in T cells on activation, and a recent CRISPR screen showed evidence that this enzyme is important in modulating T cell activation in the setting of cancer.^57^ Our findings suggest that reduced expression of B3GNT2 in individuals with the AS risk allele is likely caused by reduced chromatin openness at this locus. This may impact higher-order chromatin structures, such as the looping event identified between a distal enhancer and B3GNT2 (Figure 5). The JASPAR 2022^58^ database of TF binding profiles predicts that STAT1 binds to this site, a TF that plays an important role in transcriptional activation in the immune system.^59^ Further work, such as genomic editing and functional assays, will be needed to identify the function of these or other unknown TFs at this locus.

### Cytokine and NOTCH signaling pathways

GPCR-related and cytokine signaling pathways were consistently enriched across modalities and cell types. In particular, expression of CXC cytokine subfamily genes was upregulated in CD4^+^ and CD8^+^ T cells, consistent with the inflammatory environment of AS. Chemokine levels have been shown previously to be increased at the gene and protein level in AS^60^ and psoriatic arthritis, a related spondyloarthropathy.^61^^,^^62^ This may contribute to the differentiation of pro-inflammatory Th1 and Th17 cells, which are expanded in AS, or trafficking of leukocytes and osteoclast precursors to the sites of inflammation at the joint.^63^

NOTCH signaling has a wide range of functions in the innate and adaptive immune systems.^64^ We found that NOTCH signaling was linked to changes in chromatin signatures in monocytes. NOTCH signaling has been shown to be important for monocyte-macrophage differentiation, with increased NOTCH signaling favoring inflammatory M1 macrophage development in atherosclerosis, systemic lupus erythematosus, and cancer.^65^ NOTCH signaling has already been implicated in inflammatory states^66^ and rheumatoid arthritis^67^ and may be important for regulating monocyte differentiation to osteoclasts,^68^ thus influencing the pathogenic ossification that is a key feature of late-stage AS.^69^ Wang et al.^70^ showed that *NOTCH1* expression is reduced in AS patients who have had anti-TNF biologic therapy. Further study of this pathway in AS is warranted and could lead to repurposing of existing NOTCH pathway inhibitors in AS.^71^

### Identification of novel therapeutic targets

We showed how disease-context-specific functional genomic data could be used to identify novel therapeutic targets in AS. We modified Pi, a previously published algorithm designed to identify the network of therapeutic targets in autoimmune disease from GWAS. The multi-omics approach used here supported the importance of known candidate pathways, such as Th17/IL-23 and TNF, and identifies new pathways and potential drug targets, including PTGER4, ErbB, phosphatidylinositol 3-kinase (PI3K)/AKT, NOTCH, and GPCR (Figure 6). Existing inhibitors of these pathways could be repurposed in AS, such as PI3K inhibitors used in lymphoma treatment.^72^ Our results show exciting promise for development of new therapeutics in AS. The exact roles these TFs and other network genes play in AS remain to be elucidated and will be investigated in the future using, for example, genomic editing and functional assays.

### Conclusions

We demonstrated that the epigenomic landscape of immune cells is altered in AS. We used these results, together with evidence of chromosomal interactions, to inform the interpretation of GWASs for AS in terms of likely functional variants and modulated genes and prioritize potential drug targets and networks. This is important because existing therapies are only effective in a subset of AS patients and ultimately do not cure the disease.

### Limitations of the study

This study used disease-relevant immune cell subsets isolated from PBMCs of AS patients and HCs. One limitation of our study was the use of bulk CD4^+^ T cell, CD8^+^ T cell, and CD14^+^ monocyte populations rather than investigating smaller subsets of these cell types. This was necessary because of the large cell numbers required to perform a panel of multi-omics experiments on the same sample, in particular mapping of chromosomal interactions. We showed that major cell subsets were present at expected frequencies and did not differ between AS patients and HCs (Figure S1), although disease-specific cell type frequencies have been reported for minor cell subtypes.^20^^,^^25^^,^^26^^,^^73^ Future work sampling cells from sites of inflammation, such as sacroiliac joints, and utilizing single-cell-based methods will further unravel the cell types and pathogenic mechanisms of AS.^74^ For individual modalities, signals from differential analysis were modest, especially in CD4^+^ T cells, which could be due to the heterogeneity of this cell type. Context specificity of regulatory regions is key, so in addition to direct *ex vivo* analysis of cells from patients with the active disease state, analysis of such cells subjected to immune challenges *in vitro* (such as lipopolysaccharide stimulation of monocytes, anti-CD3/28 stimulation of T cells) may identify additional functional SNPs specifically in those activation states. The study was underpowered to perform expression and chromatin quantitative trait mapping on a genome-wide scale, and this is an important area for future work in a disease context. We were unable to study the effect of other covariates, including sex and drug regimens (although all patients were biologic therapy naive), because of the small sample size. We have shown previously that eRNAs have a role in innate immune activation.^75^ The observed widespread bidirectional eRNA expression at genomic enhancers can be further investigated using more sensitive methods (such as global run-on sequencing, small capped RNA sequencing, and precision run-on sequencing^76^) to detect more subtle alterations in eRNA expression and specifically identify the role of SNPs therein. Findings from ChromHMM were limited by small sample size. We focused on the presence of activating chromatin modifications (chromatin accessibility, promoter- and enhancer-associated histone modifications), so future studies could investigate the role of repressive histone marks such as H3K9me3 and H3K27me3. Future studies will be required to further characterize the genes and pathways highlighted by this study to assess their effect at the protein level and on cellular phenotype and function; for example, through genome editing and small-molecule inhibitors.

## STAR★Methods

### Key resources table

**Table:** 

REAGENT or RESOURCE	SOURCE	IDENTIFIER
**Antibodies**		

Anti- CD14-PE	BioLegend	Cat#325602; RRID AB_830675
Anti-CD4-BUV295	BD Biosciences	Cat# 563550, RRID:AB_2738273
Anti-CD8-BUV737	BD Biosciences	Cat#612754; RRID:AB_2870085
Anti-CD3-BV786	BD Biosciences	Cat #563918; RRID:AB_2738487
Anti-CD14-FITC	BD Biosciences	Cat #555397; RRID:AB_395798
Anti-CD16-APC	BioLegend	Cat #360706; RRID:AB_2562751
Ani-HLA-DR-BV605	BD Biosciences	Cat #562845
anti-H3K27ac	Diagenode	Cat#C15410196; RRID:AB_2637079
anti-H3K4me3	Diagenode	Cat# pAb-003-050, RRID:AB_2616052
anti-H3K4me1	Diagenode	Cat# C15410194, RRID:AB_2637078

**Biological samples**		

AS patients and HC blood samples	This study	N/A

**Chemicals, peptides, and recombinant proteins**		

Ficoll-Paque Plus	Sigma-Aldrich	GE17-1440-02
CD4 MicroBeads	Miltenyi Biotec	Cat# 130-045-101, RRID:AB_2889919
CD8 MicroBeads	Miltenyi Biotec	Cat#130-045-201; RRID:AB_2889920
CD14 MicroBeads	Miltenyi Biotec	Cat#130-050-201; RRID:AB_2665482
Trypan Blue	Gibco	15250061
FACS Lysing Solution 10x concentrate	BD Biosciences	Cat# 349202, RRID:AB_2868862
eBioScience™ Flow Cytometry Staining Buffer	ThermoFisher Scientific	Cat# 00-4222-26
7-AAD Viability Staining Solution	BioLegend	Cat# 420403
eBioScience™ 1-Step Fix/Lyse Solution	ThermoFisher Scientific	Cat# 00-5333-54
PBS, pH 7.4	Gibco	Cat#10010031
BSA	New England Biolabs	Cat#B9000S
Trizma® hydrochloride solution (Tris-HCl) pH 7.4	Sigma-Aldrich	Cat#T2194
Sodium chloride solution, 5M	Sigma-Aldrich	Cat#S5150
MgCl2 (1M)	Invitrogen	Cat#AM9530G
Digitonin	Promega	Cat#G9441
Tween 20	Sigma-Aldrich	Cat#P1379
TD Tagment DNA Buffer	Illumina	Cat#15027866
TDE1 Tagment DNA Enzyme	Illumina	Cat#15027865
AMPure XP Beads	Beckman Coulter	Cat#A63881
Formaldehyde solution	Sigma-Aldrich	Cat#F8775
Glycine	Sigma-Aldrich	Cat#G7403
Sodium dodecyl sulphate solution	Sigma-Aldrich	Cat#71736
EDTA (0.5M) pH8	Sigma-Aldrich	Cat#102161034
Tris (1 M), pH 8.0, RNase-free	Invitrogen	Cat#AM9855G
Complete Protease Inhibitor Cocktail	Sigma-Aldrich	Cat#COEDTAF-RO
Triton X-100	Sigma-Aldrich	Cat#T8787
Dynabeads Protein G for Immunoprecipitation	Invitrogen	Cat#10003D
10% Igepal CA-630	Sigma-Aldrich	Cat#I8896
DpnII 50,000 U/ml	New England Biolabs	Cat#R0543M
T4 DNA HC ligase (30 Weiss U/μL)	Thermo Fisher Scientific	Cat#EL0013
RNase, DNase free	Roche	Cat#1119915
Proteinase K	Thermo Fisher Scientific	Cat#EO0491
Dynabeads M-270 Streptavidin	Invitrogen	Cat#65305
Ribonucleic acid, transfer from baker's yeast	Sigma-Aldrich	Cat#R5636
Lithium chloride solution, 8M	Sigma-Aldrich	Cat#L7026
Sodium deoxycholate monohydrate	Alfa Aesar	Cat#B20759
Tris-EDTA buffer solution	Sigma-Aldrich	Cat#T9285

**Critical commercial assays**		

Live/Dead Fixable Violet Dead cell stain kit	Invitrogen	Cat#L34955
AllPrep DNA/mRNA/miRNA Universal kit	Qiagen	Cat#80224
Ribo-Zero rRNA Removal kit	Illumina	Cat#20040526
TruSeq Stranded Total RNA	Illumina	Cat#20020596
MinElute PCR purification kit	Qiagen	Cat#28004
NEBNext High-Fidelity 2x PCR master mix	New England Biolabs	Cat#M0541S/L
NEBNext DNA Library Prep Master Mix Set	New England Biolabs	Cat#E6040S/L
Herculase II Fusion Enzyme with dNTPs Combo	Agilent	Cat#600677
Nimblegen SeqCap EZ Hybridisation and wash kit	Roche	Cat#05634261001
Nimblegen SeqCap EZ Accessory kit v2	Roche	Cat#07145594001
KAPA Library Quantification Complete Kit (Universal)	KAPA	Cat#KK4824
NextSeq 500/550 High Output kit v2.5 (150 Cycles)	Illumina	Cat#20024907
TapeStation D1000 Screen Tape	Agilent	Cat#5067-5582
TapeStation High Sensitivity D1000 Screen Tape	Agilent	Cat#5067-5584
TapeStation D1000 Reagents	Agilent	Cat#5067-5583
Infinium Global Array V2.0	Illumina	Cat#20024444

**Deposited data**		

PCHi-C	Javierre et al.^38^	https://osf.io/u8tzp/
Ankylosing spondylitis Immunochip summary statistics	Cortes et al.^9^	N/A
Cross-disease GWAS summary statistics	Ellinghaus et al.^10^	N/A
eQTL catalogue	Kerimov et al.^43^	https://www.ebi.ac.uk/eqtl/
Human PBMC scRNA-seq data	COvid-19 Multi-omics Blood Atlas (COMBAT) consortium^77^	https://www.combat.ox.ac.uk/
RNA-seq, ATAC-seq, ChIPm fastq data	This study	European Genome-Phenome Archive: EGAS00001006233
Genotype data	This study	European Genome-Phenome Archive: EGAS00001006945
RNA-seq, ATAC-seq, ChIPm raw and normalised count data; Capture-C count data and PeakY scores; ChromHMM data	This study	https://doi.org/10.5281/zenodo.6373353

**Oligonucleotides**		

Capture-C baits	This study	Table S8
NEBNext Multiplex Oligos for Illumina (Index Primers set 1)	New England Biolabs	Cat#E7335S/L
NEBNext Multiplex Oligos for Illumina (Index Primers set 2)	New England Biolabs	Cat#E7500S/L
Nimblegen HyperCap Universal Blocking Oligos	Roche	Cat#08286396001
Modified Nextera Index primers	Buenrostro^78^	N/A

**Software and algorithms**		

CapSequm2	Telenius et al.^79^	https://capsequm.molbiol.ox.ac.uk/cgi-bin/CapSequm.cgi
CaptureCompare	Telenius et al.^79^	https://github.com/djdownes/CaptureCompare
QTLtools	Delaneau et al.^80^	https://qtltools.github.io/qtltools/
CIBERSORTx	Newman et al.^37^	https://cibersortx.stanford.edu/
Eagle2	Loh et al.^81^	https://alkesgroup.broadinstitute.org/Eagle/
PBWT	Durbin et al.^82^	https://github.com/richarddurbin/pbwt
STAR	Dobin et al.^83^	https://github.com/alexdobin/STAR/releases; RRID:SCR_004463
Picard tools	Picard Toolkit. 2019. Broad Institute, GitHub Repository.	https://broadinstitute.github.io/picard/; RRID:SCR_006525
HTSeq	Anders et al.^84^	https://htseq.readthedocs.io/en/release_0.11.1/count.html; RRID:SCR_011867
BEDTools	Quinlan et al.^85^	https://bedtools.readthedocs.io/en/latest/; RRID:SCR_006646
DESeq2	Love et al.^86^	https://bioconductor.org/packages/release/bioc/html/DESeq2.html; RRID:SCR_015687
XGR	Fang et al.^33^	http://galahad.well.ox.ac.uk:3030/
Bowtie2 v2.3.5.1	Langmead et al.^87^	http://bowtie-bio.sourceforge.net/bowtie2/index.shtml; RRID:SCR_016368
Samtools v.1.12	Danecek et al.^88^	http://www.htslib.org/; RRID:SCR_002105
bedGraphToBigWig	Kent et al.^89^	https://www.encodeproject.org/software/bedgraphtobigwig/
MACS2	Zhang et al.^90^	https://macs3-project.github.io/MACS/; RRID:SCR_013291
featureCounts	Liao et al.^91^	http://subread.sourceforge.net; RRID:SCR_012919
ChIPseeker	Yu et al.^92^	http://www.bioconductor.org/packages/release/bioc/html/ChIPseeker.html; RRID:SCR_021322
ChromHMM	Ernst et al.^39^	http://compbio.mit.edu/ChromHMM/; RRID:SCR_018141
FactoMineR	Lê et al.^93^	http://factominer.free.fr/; RRID:SCR_014602
GARFIELD	Iotchkova et al.^42^	https://www.ebi.ac.uk/birney-srv/GARFIELD/
FastQC v0.11.4	Babraham Bioinformatics	https://www.bioinformatics.babraham.ac.uk/projects/fastqc; RRID:SCR_014583
cutadapt v 1.10	Martin et al.^94^	https://cutadapt.readthedocs.io/en/stable/; RRID:SCR_011841
TrimGalore v 0.4.4	Babraham Bioinformatics	https://www.bioinformatics.babraham.ac.uk/projects/trim_galore/
FLASH	Magoč et al.^95^	https://sourceforge.net/projects/flashpage/files/FLASH-1.2.11.tar.gz/; RRID:SCR_005531
PeakY	Eijsbouts et al.^96^	https://github.com/cqgd/pky
Pi	Fang et al.^33^	http://pi.well.ox.ac.uk:3010/; ^97^
Dnet	Fang et al.^98^	https://cran.r-project.org/package=dnet
ggplot2	Wickham et al.^99^	https://ggplot2.tidyverse.org; RRID:SCR_014601
EnhancedVolcano v1.12.0	EnhancedVolcano: Publication-ready volcano plots with enhanced colouring and labelling	https://github.com/kevinblighe/EnhancedVolcano; RRID:SCR_018931
Venny 2.1	VENNY. An interactive tool for comparing lists with Venn Diagrams.	https://bioinfogp.cnb.csic.es/tools/venny/; RRID:SCR_016561
KEGG mapper – Colour	Kanehisa et al.^100^	https://www.genome.jp/kegg/mapper/color.html
GenomicInteractions	Harmston et al.^101^	https://www.bioconductor.org/packages/release/bioc/html/GenomicInteractions.html
Gviz	Hahne et al 2016^102^	https://bioconductor.org/packages/release/bioc/html/Gviz.html
Limma	Ritchie et al.^103^	https://bioconductor.org/packages/release/bioc/html/limma.html; RRID:SCR_010943
R	R Core Team (2021). R: A language and environment for statistical computing. R Foundation for Statistical Computing, Vienna, Austria	https://www.R-project.org/; RRID:SCR_001905
ICeCAP pipeline	This paper	https://doi.org/10.5281/zenodo.7760066

**Other**		

DynaMag-2 Magnet	Invitrogen	12321D
AutoMACS Pro Separator	Miltenyi Biotech	https://www.miltenyibiotec.com/GB-en/products/automacs-pro-separator-starter-kit.html#gref; RRID:SCR_018596
LSRFortessa X-20-flow cytometer	BD Biosciences	https://www.bdbiosciences.com/en-us/instruments/research-instruments/research-cell-analyzers/lsrfortessa; RRID:SCR_018655
HiSeq4000 platform	Illumina	https://emea.illumina.com/systems/sequencing-platforms/hiseq-3000-4000.html
NextSeq 500 platform	Illumina	https://emea.illumina.com/systems/sequencing-platforms/nextseq.html
M220 focussed ultrasonicator	Covaris	https://www.covaris.com/m220-focused-ultrasonicator-500295
TapeStation 4200	Agilent	https://www.agilent.com/en/product/automated-electrophoresis/tapestation-systems/tapestation-instruments/4200-tapestation-system-228263

### Resource availability

#### Lead contact

Further information and request for resources and reagents should be directed to and will be fulfilled by the lead contact, Julian Knight (julian@well.ox.ac.uk).

#### Materials availability

This study did not generate new unique reagents.

### Experimental model and subject details

#### Human subjects

20 patients with AS and 35 HC were enrolled in the study; see demographic information in Table S1. All samples were collected following informed consent and under ethical approval: National Research Ethics Service Committee South Central – Oxford Research Ethics Committee B (Ref 12/SC/0063 for patient samples, Ref 06/Q1605/55 for HC). AS patient samples were collected at the Nuffield Orthopaedic Centre in Oxford. Patients fulfilled the ASAS imaging criteria for axial Spondyloarthritis.^104^ Cases were reviewed by two consultant rheumatologists to confirm the patients had active disease at the time of recruitment as judged by the British Society for Rheumatology criteria for biologic therapy eligibility.^105^ 85% of patients and 8.6% of HC were positive for HLA-B27. All patients had axial manifestation of spondyloarthritis, and peripheral and extra-articular manifestations were present in a subset of patients. 53% of patients were taking NSAIDS, 11% were taking csDMARDs and none were taking glucocorticoids or anti-TNF biologic therapy (see clinical summary in Table S1 and extended clinical information in Table S2). All participants were aged over 18 and had no other current infections. 75% of AS patients were male, which reflects the higher frequency of disease in men; 49% of HC were male, and controls were matched for age. Human samples were collected over time and next-generation sequencing (NGS) was performed in seven sets (Table S2). NGS set and sex were included as covariates in all downstream analyses except ATAC analysis, where ATAC method and sex were included as covariates. Data quality control was performed by PCA and outliers were removed as appropriate.

### Method details

#### Isolation of immune cell populations

Up to 100 mL blood was taken from AS patients or HC into heparinised vacutainers. PBMCs were isolated from blood samples by density gradient separation using Ficoll-Paque Plus with centrifugation at 500 x *g* for 30 minutes at room temperature with minimum acceleration and no braking. Primary cell subpopulations were separated using magnetic-activated cell sorting following the manufacturer’s instructions. Consecutive positive selection was performed using beads for CD8^+^, and CD4^+^ cells using an AutoMACS Pro (Miltenyi Biotech) followed by a manual cell count with Trypan blue.

#### Flow cytometry

Cell purity following MACS separation was determined by flow cytometry. Briefly, cells were incubated with Live/Dead Fixable Violet Dead cell stain kit (Invitrogen) for 30 minutes followed by incubation with either anti-CD14-PE, anti-CD4-BUV395 or anti-CD8-BUV737 antibodies for 30 minutes at room temperature. Cells were fixed for 10 minutes using FACS Lysing Solution then re-suspended in PBS + 0.2% BSA for acquisition using LSR Fortessa X-20-flow cytometer (BD Biosciences). Cells were immediately processed for downstream assays without further culture (Figure 1A). The composition of monocyte bulk populations was determined as follows. Cells were washed with PBS and pelleted, then incubated with Human BD Fc Block™ Reagent for 10 minutes at room temperature. Cells were incubated for 30 minutes with anti-CD3-BV786, anti-CD14-FITC, anti-CD16-APC and anti-HLA-DR-BV605 antibodies diluted in eBioScience™ Flow Cytometry Staining Buffer (2 μL antibody/1x106 cells). Cells were also stained for viability using 7-AAD Viability Staining Solution then fixed for 10 minutes with 1X eBioScience™ 1-Step Fix/Lyse Solution, and washed with 0.2% PBS-BSA prior to flow cytometry analysis using a BD LSRFortessa™ X-20 Cell Analyzer. 1x105 events were recorded for each sample.

#### RNA-seq

Total RNA was isolated from purified CD4^+^ T cells, CD8^+^ T cells and monocytes (2-3 x 10^6^ cells resuspended in RLT Plus) using the AllPrep DNA/mRNA/microRNA Universal kit (Qiagen) from 16 AS patients and 19 HC. Samples were depleted from ribosomal RNA using Ribo-Zero rRNA Removal kit (Illumina) prior to cDNA synthesis and library preparation using TruSeq Stranded Total RNA (Illumina). Libraries were sequenced using HiSeq4000 to a depth of 25 million paired-end reads per sample.

#### ATAC-seq

ATAC-seq was used to assess chromatin accessibility in CD4^+^ T cells, CD8^+^ T cells and monocytes from 16 AS patients and 30 HC. Three different variations of the ATAC-seq protocol were used as improvements to the method were published: ATAC-seq^106^ (5 x 10^4^ cells); Fast-ATAC^107^ (FATAC, 2 x 10^4^ cells); Omni-ATAC^108^ (OATAC, 5 x 10^4^ cells), with minor modification to determine amplification by using 10% of the sample in qPCR prior to indexing and amplification. Following MACS isolation, ATAC was carried out. For ATAC-seq cells were re-suspended in 50 μL lysis buffer (10 mM Tris-HCl, pH 7.4, 10 mM NaCl, 3 mM MgCl2, 0.1% IGEPAL CA-630) for 10 minutes at 4°C. Nuclei were pelleted (500 x g 10 min at 4°C) and re-suspended in 50 μL transposition mixture (1 x TD Tagment DNA buffer (Illumina), 2.5 μL TDE1 (Illumina) and incubated for 40 min at 37 °C with agitation at 400 rpm. For Fast-ATAC-seq cells were incubated in 50 μL transposition mixture (1 x TD Tagment DNA buffer (Illumina), 2.5 ul of TDE1 (Illumina), 0.01% Digitonin) for 30 minutes at 37°C with agitation at 400 rpm. For Omni-ATAC-seq cells were re-suspended in 50 μL lysis buffer (10 mM Tris-HCl pH7.4, 10 mM NaCl, 3 mM MgCL2, 0.01% Digitonin, 0.1% Tween 20, 0.1% Igepal CA-630) for 3 minutes at 4°C, then 1 mL cold wash buffer (10 mM Tris-HCl pH7.4, 10 mM NaCl, 3 mM MgCL2, 0.1% Tween 20) was added. Nuclei were pelleted (500 x g 10 min at 4°C), re-suspended in 50 μL transposition mixture (1 x TD Tagment DNA buffer (Illumina), 2.5 μL TDE1 (Illumina), 0.01 % Digitonin and 0.1 % Tween-20), and incubated for 30 min at 37°C with agitation at 1000 rpm. DNA was extracted with MinElute PCR Purification Kit (Qiagen). Samples were amplified and indexed as in Buenrostro et al^109^ with NEBNext High-Fidelity PCR MasterMix (NEB) with modified Nextera indexing primers.^78^ DNA libraries were purified using the MinElute PCR purification kit (Qiagen) and AMPure XP Magnetic Beads (Beckman Coulter). Library distribution was determined using TapeStation D1000 reagents and tape. Libraries were sequenced on a HiSeq4000 or NextSeq500 Illumina platform to a depth of 30 million paired-end reads after filtering.

#### ChIPmentation

ChIPmentation (ChIPm) for H3K4me3 and H3K27ac was performed as described^110^ on samples of 1x10^5^ CD4^+^ T cells, CD8^+^ T cells and monocytes from 10 AS patients and 11 HC.

ChIPm was also performed for H3K4me1 on samples from one healthy individual not included in the other experiments. Following MACS isolation, cells were fixed with 1% formaldehyde (Sigma) in PBS for 10 min, then quenched with glycine (0.25 M for 5 min). Cells were pelleted (100 x g 2.5 minutes), washed with PBS, then pelleted again and re-suspended in 130 μL SDS lysis buffer (0.25% SDS, 1mM EDTA, 10mM Tris-HCl pH8, 1x Protease Inhibitor) and sonicated for 8 min using M220 Focused-ultrasonicator (Covaris), duty factor 10%, peak power of 75, cycles/burst of 200 set to 7°C. 50 μL chromatin aliquots were mixed with 75 μL ChIP Equilibration Buffer (1.66% Triton-X100, 1 mM EDTA, 10 mM Tris-HCl pH8, 233 mM NaCl, 1x protease inhibitor) and 25 μL ChIP buffer (0.1 % SDS, 1% Triton-X100, 1 mM EDTA, 10 mM Tris-HCl pH8, 140 mM NaCl, 1x protease inhibitor). Antibodies (1 μg anti-H3K4me3, 2 μg anti-H3K27ac, 1 μg anti-H3K4me1) were added and incubated overnight at 4 °C with rotation. Protein G Dynabeads were prepared in bead wash buffer (0.1% SDS, 1 mM EDTA, 50 mM Tris-HCl pH8, 150 mM NaCl, 1% Igepal CA-630, 1 x protease inhibitor) and blocked with yeast tRNA and BSA as per the manufacturer’s instructions. Samples and beads were incubated for 2 hr 4°C with rotation, then washed twice with 200 μL each wash buffer (wash buffer 1: 0.1% SDS, 1% Triton-X100, 2 mM EDTA, 20 mM Tris-HCl pH8, 150 mM NaCl, 1 x protease inhibitor; wash buffer 2: 0.1% SDS, 1% Triton-X100, 2 mM EDTA, 20 mM Tris-HCl pH8, 500 mM NaCl, 1 x protease inhibitor; wash buffer 3: 0.25 M LiCl, 1% Triton-X100, 0.7% sodium deoxycholate monohydrate, 1 mM EDTA, 10 mM Tris-HCl pH8, 1 x protease inhibitor) and once with 10 mM Tris-HCl pH8. Samples were finally resuspended in 20 μL Tagmentation reaction buffer (1 x TD buffer, 1 μL TDE 1 Tagment DNA Enzyme (Illumina)) and incubated for 10 min at 37 °C. Samples were washed twice with 200 μL wash buffer (0.1% SDS, 1% Triton-X100, 2 mM EDTA, 20 mM Tris-HCl pH8, 150 mM NaCl, 1 x protease inhibitor) and once with Tris-EDTA buffer. Samples were de-crosslinked in ChIP Elution buffer (10 mM Tris-HCl pH8, 5 mM EDTA, 300 mM NaCl, 0.4% SDS) containing 2.4 U proteinase K for 1 hour at 55 °C then overnight at 65 °C with 1400 rpm shaking. Samples were purified using the MinElute PCR purification kit (QIAgen). Indexing was performed with modified Nextera indexing primers^78^ and NEBNext HiFi PCR master mix using cycle number determined by qPCR. PCR product clean-up was performed using AMPureXP beads and QC was performed using TapeStation D1000 tape and reagents. DNA quantitation was performed using KAPA assay. Input libraries were prepared using 1 ng of purified chromatin without antibody incubation. Libraries were sequenced using the HiSeq4000 Illumina platform at a depth of 25 million paired-end reads after filtering.

#### Capture-C

Capture-C experiments were performed as described by Davies et al^111^ with minor changes.

Oligonucleotide baits for sequence-capture were designed for regions containing (i) 52 GWAS SNPs associated with AS,^9^^,^^10^ (ii) 47 promoters of nearby genes, and (iii) five control regions with known genomic interactions (Table S8, Figure S14). Two biotinylated 120nt ssDNA bait sequences were designed for each viewpoint (DpnII fragment to be captured) using CapSequm2^79^ and synthesized by Sigma-Aldrich. Some bait oligos were 80nt and some baited viewpoints had only one oligo bait due to regional sequence repetitiveness. All oligo baits were combined in equimolar amounts to make a pool containing each oligo at 2.9nM. For two regions, interactions were seen not with the baited promoter, but with other genes so results are presented with those gene names (*ITGA4* relates to bait for *UBE2E3*, and *IL12RAP* relates to bait for *IL1R2*). We were unable to capture the DpnII fragment containing rs4672505 using Capture-C due to DNA repeats.

Following MACS isolation, 10-30 x 10^6^ of monocytes, CD8^+^ T cells and CD4^+^ T cells from three AS patients and three HC were fixed with 2% formaldehyde (10 min), quenched with 0.1M glycine (10 min on ice), washed with PBS and snap frozen in 1ml of lysis buffer (10 mM Tris-HCl pH8, 10 mM NaCl, 0.3% Igepal CA-630, 1 x protease inhibitor). Cells were thawed and centrifuged at 500 x *g* for 5 minutes at RT. The supernatant was removed, cells were washed in 1 mL of 1 x DpnII buffer (NEB) to remove the residual lysis buffer and spun down at 500 x *g* for 5min at room temperature. Cells were re-suspended in 200 μL of 1 x DpnII buffer per every 6 x 10^6^ cells and homogenized to free the nuclei. Chromatin was digested using 1500U of DpnII per 6 x 10^6^ cells overnight at 37 °C with shaking 900 rpm; the reaction was stopped by incubation for 20 minutes at 65 °C. Ligation was performed with 240 U T4 DNA ligase overnight at 16 °C. Controls were prepared without DpnII digestion (Undigested) and without ligation steps (Digested). De-crosslinking was performed with addition of 3 U Proteinase K and overnight incubation at 65 °C. Samples were treated with RNase A for 30 min at 37 °C then DNA was purified by phenol-chloroform extraction and ethanol precipitation. Covaris M220 focussed ultrasonicator was used to fragment the 3C material to 200 bp (duty cycle, 20%; intensity, 50; cycles per burst, 200; time, 280s), then AMPure XP SPRI bead cleanup was performed. Illumina TruSeq sequencing adapters (NEBNext Multiplex Oligos Index Primers sets 1 and 2, NEB) were added to 5μg of sonicated 3C material using NEBNext DNA Library Prep Master Mix Set (NEB) reagents for end repair, dA tailing and adaptor ligation, Herculase II Fusion Enzyme (Agilent) for indexing PCR, and AMPure® XP Beads (Beckman Coulter) for clean-up steps.

Selective enrichment of 3C libraries was performed as described^111^ with minor changes using Nimblegen SeqCap EZ Hybridisation and wash kit, Accessory kit v2 (Roche) and their HyperCap Workflow v2.0.

Briefly, the oligo bait pool was hybridised to a pool of indexed 3C libraries (up to six 3C libraries equating to 6 μg of material per hybridisation reaction) in the presence of Universal Blocking Oligos (Roche) and SeqCap EZ reagents (Roche) at 47°C for 72h. The captured fragments were pulled down with M-270 Streptavidin Dynabeads (Invitrogen), washed with SeqCap EZ wash buffers, cleaned up using AMPure XP Beads (Beckman Coulter) and amplified using KAPA Library Quantification Complete Kit (Roche) (9-12 cycles). The resulting enriched library was used as an input for a secondary capture following the same protocol as above, but with hybridization time of 24h and fewer final PCR cycles determined by a test qPCR reaction. Quality control was performed using TapeStation high sensitivity D1000 screen tape and reagents. Capture-C libraries were sequenced on the Illumina NextSeq 500 platform using 150bp paired-end reads at a depth of 1 million paired end reads per viewpoint.

#### Genotyping

DNA was isolated from purified CD4^+^ T cells, CD8^+^ T cells and monocytes (2-3 x 10^6^ cells resuspended in RLT Plus) using the AllPrep DNA/mRNA/microRNA Universal kit (Qiagen) from 20 AS patients and 20 HC. Samples were submitted for genome wide array genotyping at the Oxford Genomics Center and processed using the Infinium Global Array V2.0 (Illumina).

### Quantification and statistical analysis

#### Analysis of flow cytometry data

Flow cytometry data were analysed using FlowJo Version 10.8.1. Cells were gated by size (FSC) and granularity (SSC) and then for singlets by FSC-H vs FSC-W followed by SSC-H vs. SSC-W. CD3^neg^ cells were selected using CD3 vs. SSC-A. A CD14 vs. CD16 plot was used to define CD14hi/CD16neg classical monocytes (cMono), CD14hi/CD16pos intermediate monocytes (iMono), and CD14lo/CD16pos non-classical monocytes (ncMono). CD14lo/CD16pos cells were defined as non-monocytic (not Mono).

#### Downstream analysis of NGS data

##### RNA-seq

NGS data was mapped to human genome assembly GRCh37 (hg19) using STAR,^83^ reads were counted using featureCount^91^ with those mapping to X and Y chromosomes removed. Duplicates were marked and removed using Picard Tools. Genes lying within 500kb of lead AS-associated SNPs^9^^,^^10^ were identified using BEDtools^85^ window. PCA was performed using DESeq2 accounting for sex and sequencing set using limma^103^ (Table S2). Differential analysis between AS patients and HC was performed using DESeq2^86^ with sex and batch effect included in the design. Thresholds padj <0.05 and fold-change >1.5 were used to call significantly differential genes. Enriched pathways were identified in REACTOME pathways using XGR.^33^

##### ATAC-seq, ChIPm and eRNA analysis

Reads were aligned to the human genome assembly GRCh37 (hg19) using bowtie2.^87^ Picard Tools was used to remove PCR duplicates, read with MAPQ score <30, non-uniquely mapping reads, non-properly paired reads and mitochondrial reads. Pileup tracks were generated using BEDTools genomCoverageBed and bedGraphToBigWig.^85^ Normalised bigWigs were generated from normalised bedgraph files with BEDTools genomecov. Peak calling was performed using MACS2 callpeak^90^ and peak master lists were built by union of peaks present in at least 20% of samples. Reads for ATAC and ChIPm were counted using HTSeq,^84^ with those from X and Y chromosomes removed subsequently. Enhancer RNAs (eRNAs) were defined as uniquely mapped RNA reads within ATAC-seq peaks > 3kb from a gene coding sequence and were counted using featureCounts.^91^ Genomic distributions of ATAC peaks, ChIP peaks and eRNAs were generated using ChIPseeker.^92^ In all cases features were filtered that did not have at least 10 reads in the smallest batch, or in at more than one sample for ChIP. PCA was performed on ATAC-seq, ChIPm and eRNA data both across and within each cell type using DEseq2^86^ accounting for sex and batch using limma.^103^ Differential analysis within each cell type to compare samples from AS patients and HC was performed using DESeq2^86^ including sex and batch in the design. QTLtools^80^ was used for QTL discovery and trait significance was calculated using ANOVA with Tukey post-test.

#### Computational deconvolution of bulk RNA-seq data

The composition of CD4^+^ T cell, CD8^+^ T cell and monocyte populations was analysed by deconvolution of bulk RNA-seq data using CIBERSORTx^37^. Reference single-cell RNA-seq data from PBMC from 10 healthy individuals was obtained from the COMBAT consortium.^77^ Raw counts for cells annotated as CD4, CD8, or monocyte (comprising cMono or ncMono) were extracted and used as inputs for the CIBERSORTx Create Signature Matrix function, performed with parameters Min.Expression = 0.25, Replicates = 20 and Sampling = 0.5. The CIBERSORTx Impute Cell Fractions module was run using these Signature Matrices and raw bulk RNA-seq counts from the CD4^+^ T cell, CD8^+^ T cell and CD14^+^ monocyte populations with S-mode batch correction and 100 permutations. Annotations were used according to the COMBAT consortium minor subsets. For visualisation, cycling classical monocytes (cMono_cyc) were merged with classical monocytes (cMono), proliferating T Effector cells (TEFF.prolif) were merged with T effector cells (TEFF), and CCL5-expressing T central memory cells (TCM_CCL5) were merged with T central memory cells (TCM).

#### ChromHMM

ChromHMM^39^ was run on a subset of four AS patients and four HC samples for which we had all data types, RNA, ATAC, H3K4me3 and H3K27ac and were processed together as a single batch. A generic H3K4me1 track was used that was independent of all samples. For the coding track a binarized file of the genes was generated based on the presence or absence of the gene within each 200bp bin. The default settings were used for ChromHMM and a 14-state model based on analysis of the output for varying model sizes. Manual curation was used to assign putative functions to each state according to the combination of epigenomic marks and genomic distribution. MCA was performed on global or individual chromatin states using FactoMineR.^93^ In order to reduce the number of regions analysed (to reduce computational time), the most polymorphic regions were selected. For the global analysis (Figure 3D), 200-bp regions were kept if they contained at least two different states, and if the quiescent state 14 (Quies) was present in less than ⅔ of the samples. For individual state analysis (Figure S3B), regions were selected where that state was present in at least 1/3 of the samples.

Differences in ChromHMM states between AS patients and HC were assessed by Fisher exact tests for each state and each cell type separately. Because of the limited sample size (4 AS, 4 HC) and of the binary parameter analysed, the number of p-values obtained was limited with a minimal p value of 0.05 corresponding to a state present in 4 AS patients and no HC or 4 HC and no AS patients. To identify states with a higher number of differentially distributed regions between the two groups than expected by chance, we permuted the disease status as many times as possible (70 combinations) and compared the experimentally observed frequency of significant regions (Fisher p < 0.05) with the permuted distribution (Figure S3B). From this we calculated a p value whose significance is reported in Figure 3C. The most significant p value possible is 1/70 i.e. 0.015.

#### Pathway enrichment analysis

ATAC and ChIP peaks, eRNAs and ChromHMM regions were assigned to genes on the basis of (i) genomic proximity and (ii) genomic looping events. Proximal genes (<50 kb) were annotated using XGR.^36^ Genomic looping events were obtained from PCHi-C data^38^ from the cell types most similar to those in our study (total CD4^+^ and CD8^+^ T cells, and monocytes). Regions with >50% overlap with the ends of PCHi-C loops were identified using BEDtools.^85^ Pathway enrichment analysis in REACTOME and KEGG databases was performed using XGR on gene sets from individual cell types. Gene sets were generated from the top 200 differential ATAC-seq peaks, ChIPm peaks or eRNAs, and genes associated with differential ChromHMM regions (Fisher p < 0.05).

#### GWAS enrichment

Enrichment of AS-associated variants was determined using GARFIELD^42^ for each of the epigenomic marks identified in this study (ATAC, H3K27ac and H3K4me3) and the chromatin states defined by ChromHMM analysis. Enrichment of AS variants from European subanalysis of Cortes et al.^9^ was assessed at four GWAS significance thresholds: p < 1, 0.1, 5 × 10−7 and 5 × 10−8. The UK10K variant set,^112^ pre-processed in GARFIELD, was used as the reference population for these analyses, with correction for multiple testing performed using the Bonferroni method.

#### Capture-C NGS data analysis

The quality of Illumina reads was validated with FastQC v0.11.4 and the presence of Illumina adapter sequences addressed using cutadapt v1.10.^94^ TrimGalore v.0.4.4 was used to automate quality and adaptor trimming. We extended the length of the pair-end libraries following the overlap and merge criteria of FLASH96 a fast length adjustment algorithm 100, so that two sets of merged and non-merged reads were generated for downstream analysis.

A single core version of FLASH was modified for Capture-C analysis, to allow for in-silico digestion of flashed and non-flashed reads at the DpnII recognition sequence, subsequent to the merging step. Pairs of in-silico digested, reported read sequences were enumerated, counted and aligned to hg19 using bowtie2.^87^ All in-silico digested sub-sequence pairs were assessed and the corresponding mapping quality reported, together with the genomic location of the pair ends, within both flashed and non-flashed read set independently. Any pair containing sub-reads shorter than a minimum 20 base pairs were discarded. All pairs that passed this stage were then considered for downstream analysis. Duplicated pairs were enumerated and excluded using samtools.^113^ Pairs containing low quality mapping reads (Phred Quality Score <30) on either end were also excluded. De-duplicated valid paired-end reads were then considered for bona-fide Capture-C filtering, using an in-house algorithm, ICeCAP (see key resources table). In brief, after flashing and aligning reads and after in-silico digestion, artefacts, e.g. self-ligation and re-ligation events were identified, enumerated and excluded based on the principles of the HiCUP pipeline. Remaining fragments were allocated to each bait and grouped in promoter specific and enhancer specific bona-fide ditags for statistical analysis. ICeCAP combines the principles of fast length adjustment (FLASH) and bona-fide Capture-HiC filtering principles of the HiCUP pipeline. The significance of interaction frequencies was assessed using Peaky^96^. CaptureCompare^79^ was used to compare AS and HC scores and to generate mean tracks for visualisation. Median PeakY interaction scores were calculated from the top 10% of scores across the region of interest, which were defined based on ChromHMM enhancer/promoter delineation. *ETS1* promoter region chr11:128310000-128410000, *ETS1* SNP region chr11:128150000-128185000. *PTGER4* promoter region chr5:40650000-40690000, rs1992661 SNP region, chr5:40370000-40420000, rs9283753 SNP region chr5:40480000-40505000.

#### Genetic analysis and imputation

Data QC was performed by the Oxford Genomics Centre using Genome Studio v2.0 (Illumina, Human genome reference assembly GRCh37/hg19). To impute B27 from the genotyping data, we used snp2hla on chromosome 6 of genotyped data using the T1DGC reference panel, as described in Jia et al.^114^ For genome wide imputation SNPs were removed if they had a minor allele frequency (MAF) < 5%, missingness >2% or a heterozygosity rate greater than 3 standard deviations from the mean. We also removed SNPs if they deviated from Hardy-Weinberg Equilibrium (p < 1x10^−7^). Imputation was performed on the Sanger Imputation Server with the HRC reference panel^115^ using Eagle2^81^ and PBWT.^82^ After imputation, we kept SNPs with an info score >0.8, MAF >0.05 and in Hardy Weinberg equilibrium.

#### Therapeutic target prioritisation

Using our previously established Pi pipeline,^33^ we first prepared three types of genomic predictors taking as inputs GWAS summary data in AS^9^ and the knowledge of protein interactions^116^ including: (i) nearby genes (the *nGene* predictor) using genomic proximity and organisation^117^ (ii) expression-associated genes (the eGene predictor) integrating eQTL datasets,^118^^,^^119^^,^^120^^,^^121^^,^^122^ and (iii) conformation genes (the cGene *predictor*) using PCHi-C datasets.^38^ Next, we modified the Pi algorithm to prepare additional five types of genomic predictors using disease-specific functional genomic datasets arising from this study, including (iv) the AS-specific expression predictor (the *RNA* predictor) generated based on differential gene expression between AS patients and HC, and (v) AS-specific epigenomic predictors (the *ATAC*, *eRNA*, *H3K4me3* and *H3K27ac* predictors) on the basis of differential peaks (linked to genes via genomic proximity or PCHi-C looping events) between AS patients and HC. The knowledge of protein interactions was obtained from the STRING database,^116^ and only the genes located in chromosomes 1-22 were considered; this corresponded to a total of 17,249 genes prioritised (and ranked by priority rating). The performance benchmarking (including how to define clinical proof-of-concept targets and simulate negative targets) and comparisons with naïve prioritisation and Open Targets^49^^,^^50^ were the same as previously described^33^^,^^34^ and uses Area Under the Curve (AUC) as a global measure of performance. We carried out pathway enrichment analysis of the top 1% prioritised genes using KEGG pathways^123^ with the enrichments measured by Z-score and FDR (one-sided Fisher’s exact test). Using the algorithm originally proposed in dnet,^98^ we performed pathway crosstalk analysis to identify a subset of gene interactions (merged from KEGG pathways) that contained highly prioritised and interconnecting genes.

#### Data visualisation

Results from PCA, gene expression, pathway analysis and GWAS enrichment were plotted with ggplot2. Volcano plots were generated with EnhancedVolcano. The Venn diagram showing cell type specificity of differentially expressed genes was generated using Venny. Colour mapping of gene expression (by fold-change) in the CXC Cytokine Receptor pathway was performed using KEGG mapper – color pathway.^100^

Genomic data including location of ENSEMBL genes, ChromHMM data, RNA-seq, ATAC-seq data, ChIPm data, PCHiC data, and Capture-C interactions were plotted in R (https://www.R-project.org/) using Gviz^102^ and GenomicInteractions.^101^ For visualisation RNA-seq was plotted in bins of 1000bp using the log2 of the count. ATAC-seq and ChIPm-seq were visualised with sliding windows of 200bp and 400bp respectively. Capture-C tracks are plotted as mean counts from three AS patients and three HC samples in bins of 400bp, with the PeakY interaction score (-log10(FDR)) plotted below each pile-up track with a sliding window of 400bp. PCHiC interactions were plotted with a CHiCAGO^124^ score of >=5. For ATAC-seq and ChIPm tracks, data from one representative AS patient and one HC are shown. For ChromHMM data from all four AS patients and controls is shown.

## Data Availability

• Sequence level RNA-seq, ATAC-seq, ChIPm fastq and genotype data have been deposited at the European Genome-Phenome Archive (EGA); access is managed by a Data Access Committee. Count data for RNA-seq, ATAC-seq, ChIPm and eRNA (raw and normalised), Capture-C mean pile-up tracks and PeakY scores, and ChromHMM data are deposited at Zenodo. Accession numbers are listed in the key resources table. This paper analyses existing, publicly available data (GWAS data, PCHiC data and eQTL). The accession numbers for the datasets are listed in the key resources table. • All original code for the ICeCAP pipeline has been deposited at GitHub and Zenodo and is publicly available as of the date of publication. DOIs are listed in the key resources table. • Any additional information required to reanalyse the data reported in this paper is available from the lead contact upon request.

## Supplemental information

Document S1. Figures S1–S15 and Table S1

Table S2. Experiments performed on samples from each individual and extended clinical data, related to Table S1 and STAR Methods AS patients and HC are listed. Set is the NGS sequencing set (used for batch correction in downstream NGS data analysis). Three ATAC methods are as described in STAR Methods. ∗ indicates a subset of OATAC samples that were processed as a separate batch due to technical variation in sample preparation. X indicates whether each experiment (RNA-seq, ATAC, ChIPm), ChromHMM analysis, or genotyping was performed using samples from each individual in each cell type. Sex and age range are given for all individuals in the study. The following clinical features are documented for AS patients: HLA-B27 status, disease duration (months), Bath Ankylosing Spondylitis Disease Activity Index (BASDAI), Ankylosing Spondylitis Disease Activity Score with CRP (ASDAS-CRP) and C-reactive protein (CRP) (mg/L) at time of recruitment, presence of comorbidities, family history of AS and related conditions, and drug treatments. CRP was not measured for patients AS201-207, so CRP and ASDAS-CRP are not available; ASDAS-CRP not calculated for AS018 due to missing clinical scores.

Table S3. Differentially expressed genes between AS patients and HCs in CD4^+^ T cells, CD8^+^ T cells, and monocytes, related to Figure 1 Output of DEseq2 analysis, significant genes defined as padj <0.05 and FC > 1.5. Spreadsheet sheet name shows the cell type. EnsemblID, Ensembl gene identified; baseMean, normalized count values; log2FoldChange, log2 FC of AS vs. HC; lfcSE, standard error of log2FoldChange; stat, Wald statistic; pvalue, Wald test p-value; padj, Benjamini-Hochberg adjusted p-value; name, gene name.

Table S4. Differential ATAC-seq peaks between AS patients and HCs in CD4^+^ T cells, CD8^+^ T cells, and monocytes, related to Figure 2 Output of DEseq2 analysis, significant peaks defined as padj <0.05 and FC > 1.5. Spreadsheet sheet name shows the cell type. Peak_location, position of peak; baseMean, normalized count values; log2FoldChange, log2 FC of AS vs. HC; lfcSE, standard error of log2FoldChange; stat, Wald statistic; pvalue, Wald test p-value; padj, Benjamini-Hochberg adjusted p-value; ProxGene, nearest coding gene; Dist, distance to nearest coding gene.

Table S5. Differential ChIP-seq peaks between AS patients and HCs in CD4^+^ T cells, CD8^+^ T cells, and monocytes, related to Figure 2 Output of DEseq2 analysis for H3K4me3 and for H3K27ac, significant peaks defined as padj <0.05 and FC > 1.5. Spreadsheet sheet name shows the cell type. Peak_location, position of peak; baseMean, normalized count values; log2FoldChange, log2 FC of AS vs. HC; lfcSE, standard error of log2FoldChange; stat, Wald statistic; pvalue, Wald test p-value; padj, Benjamini-Hochberg adjusted p-value; ProxGene, nearest coding gene; Dist, distance to nearest coding gene.

Table S6. Differentially expressed eRNAs between AS patients and HCs in CD4^+^ T cells, CD8^+^ T cells, and monocytes, related to Figure 2 Output of DEseq2 analysis, significant eRNAs defined as padj <0.05 and FC > 1.5. Spreadsheet sheet name shows the cell type. Peak.name, position of eRNA; baseMean, normalized count values; log2FoldChange, log2 FC of AS vs. HC; lfcSE, standard error of log2FoldChange; stat, Wald statistic; pvalue, Wald test p-value; padj, Benjamini-Hochberg adjusted p-value; ProxGene, nearest coding gene; dist, distance to nearest coding gene.

Table S7. Differential ChromHMM regions between AS patients and HCs in monocytes, related to Figure 3 Differential ChromHMM regions (p < 0.05 Fisher exact test) in monocytes. Region_name, position of differential region; size, size of differential fragment in bp; AS, presence in number of AS patients; HC, presence in number of HC; loop_name, position of interacting PCHi-C loop and interaction score; loop_gene, name of interacting gene (“.” if none); ProxGene, nearest proximal gene <50 kb; ProxGene_Dist, distance to proximal gene in bp; state, ChromHMM state as Figure 3B.

Table S8. Sequence and co-ordinates of Capture-C bait oligonucleotides, related to Figure 5 Control viewpoints are named GENE_control. Promoter viewpoints are named GENE_Pro. SNP viewpoints are named GENE_SNP. Two oligonucleotides were designed per viewpoint. 5Biosg indicates 5′biotin modification.

Document S2. Article plus supplemental information
