# Supplementary material for: Comprehensive epigenomic profiling reveals the extent of disease-specific chromatin states and informs target discovery in ankylosing spondylitis
Source: Cell Genom. 2023 Apr 24;3(6):100306. doi: 10.1016/j.xgen.2023.100306 (PMC10300554; doi:10.1016/j.xgen.2023.100306)
Supplement: Document S1. Figures S1–S15 and Table S1 [file mmc1.pdf]

Cell Genomics, Volume *3*

Supplemental information

Comprehensive epigenomic profiling reveals the extent of disease-specific chromatin states and informs target discovery in ankylosing spondylitis

Andrew C. Brown, Carla J. Cohen, Olga Mielczarek, Gabriele Migliorini, Félicie Costantino, Alice Allcock, Connor Davidson, Katherine S. Elliott, Hai Fang, Alicia Lledó Lara, Alice C. Martin, Julie A. Osgood, Anna Sanniti, Giuseppe Scozzafava, Matteo Vecellio, Ping Zhang, Mary Helen Black, Shuwei Li, Dongnhu Truong, Julio Molineros, Trevor Howe, B. Paul Wordsworth, Paul Bowness, and Julian C. Knight

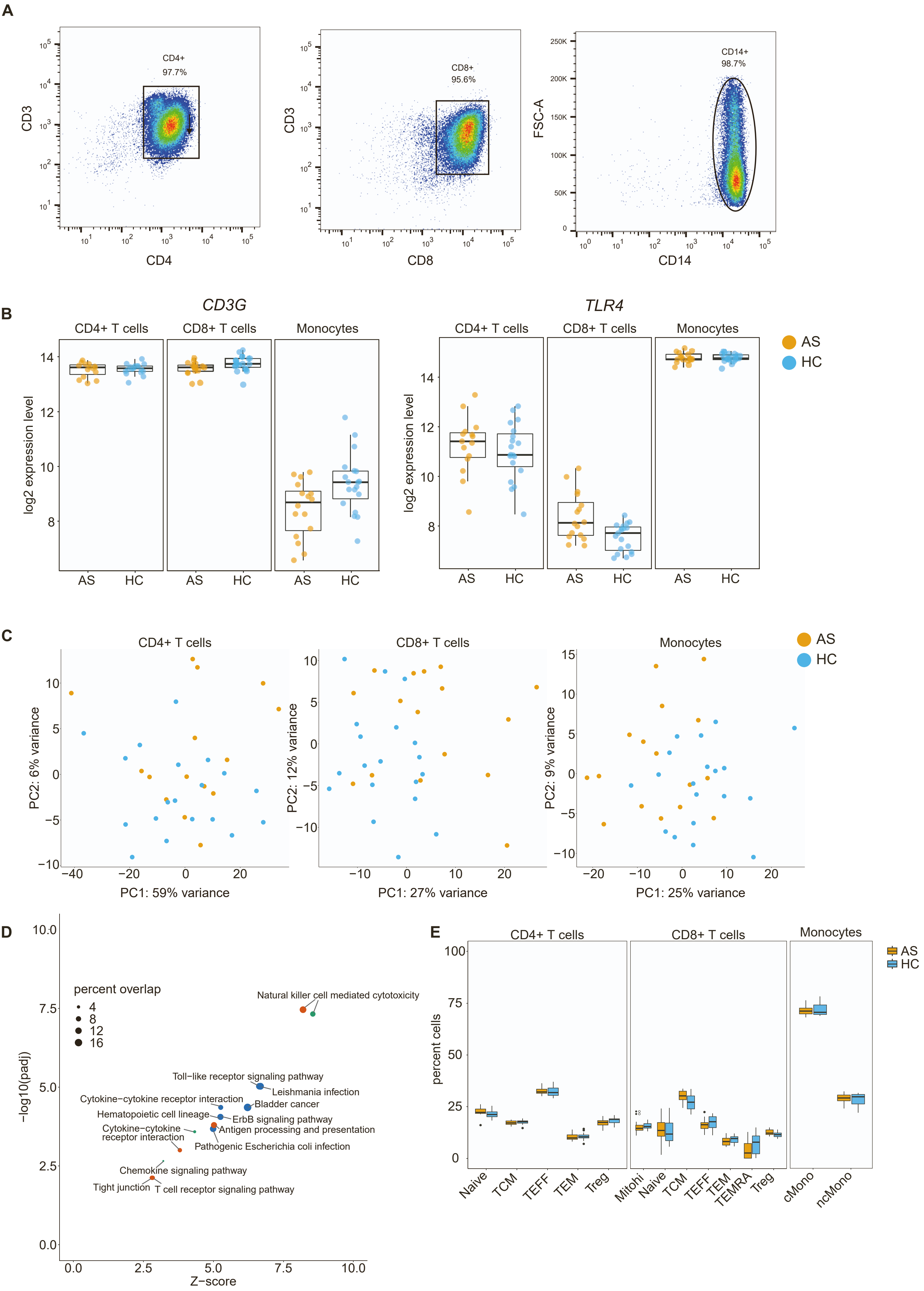
A
CD4+
97.7%
CD3
CD4
CD8+
95.6%
CD3
CD8
CD14+
98.7%
FSC-A
CD14
B
CD3G
TLR4
CD4+ T cells
CD8+ T cells
Monocytes
log2 expression level
AS
HC
C
CD4+ T cells
CD8+ T cells
Monocytes
PC2: 6% variance
PC1: 59% variance
PC2: 12% variance
PC1: 27% variance
PC2: 9% variance
PC1: 25% variance
AS
HC
D
percent overlap
4
8
12
16
Natural killer cell mediated cytotoxicity
Toll−like receptor signaling pathway
Leishmania infection
Cytokine−cytokine receptor interaction
Bladder cancer
Hematopoietic cell lineage
ErbB signaling pathway
Cytokine−cytokine receptor interaction
Antigen processing and presentation
Pathogenic Escherichia coli infection
Chemokine signaling pathway
Tight junction
T cell receptor signaling pathway
−log10(padj)
Z−score
E
CD4+ T cells
CD8+ T cells
Monocytes
AS
HC
percent cells
Naive
TCM
TEFF
TEM
Treg
Mitohi
Naive
TCM
TEFF
TEM
TEMRA
Treg
cMono
ncMono

Figure S1: Cell-type specific gene expression highlights differential genes and pathways in AS. Related to Figure 1.

(A) Purity of CD4+ T cells, CD8+ T cells and monocytes following isolation from PBMC. Cells were stained with antibodies against CD3 in combination with CD4 (left), CD8 (centre) or CD14 (right) and analysed by flow cytometry to demonstrate >95% purity of isolated cell populations.

(B) Examples of cell type-specific gene expression from RNA-seq analysis (TLR4 highly expressed in in monocytes and CD3G in T cells).

(C) PCA showing distribution of gene expression in AS patients and HC in each cell type.

(D) Enriched pathways in KEGG database (padj < 0.01 from XGR output) from significant differentially expressed genes in each cell type. Size of dot represents percentage of genes represented in that pathway, colours represent cell types.

(E) Bulk cell population composition of CD4+ T cells, CD8+ T cells and monocytes determined via deconvolution of bulk RNA-seq data using CIBERSORTx. T central memory, TCM; T effector, TEFF; T effector memory, TEM; Regulatory T cell, Treg; High mitochondrial content, mitohi; T effector memory expressing CD45RA, TEMRA; classical monocytes, cMono; non-classical monocytes, ncMono.

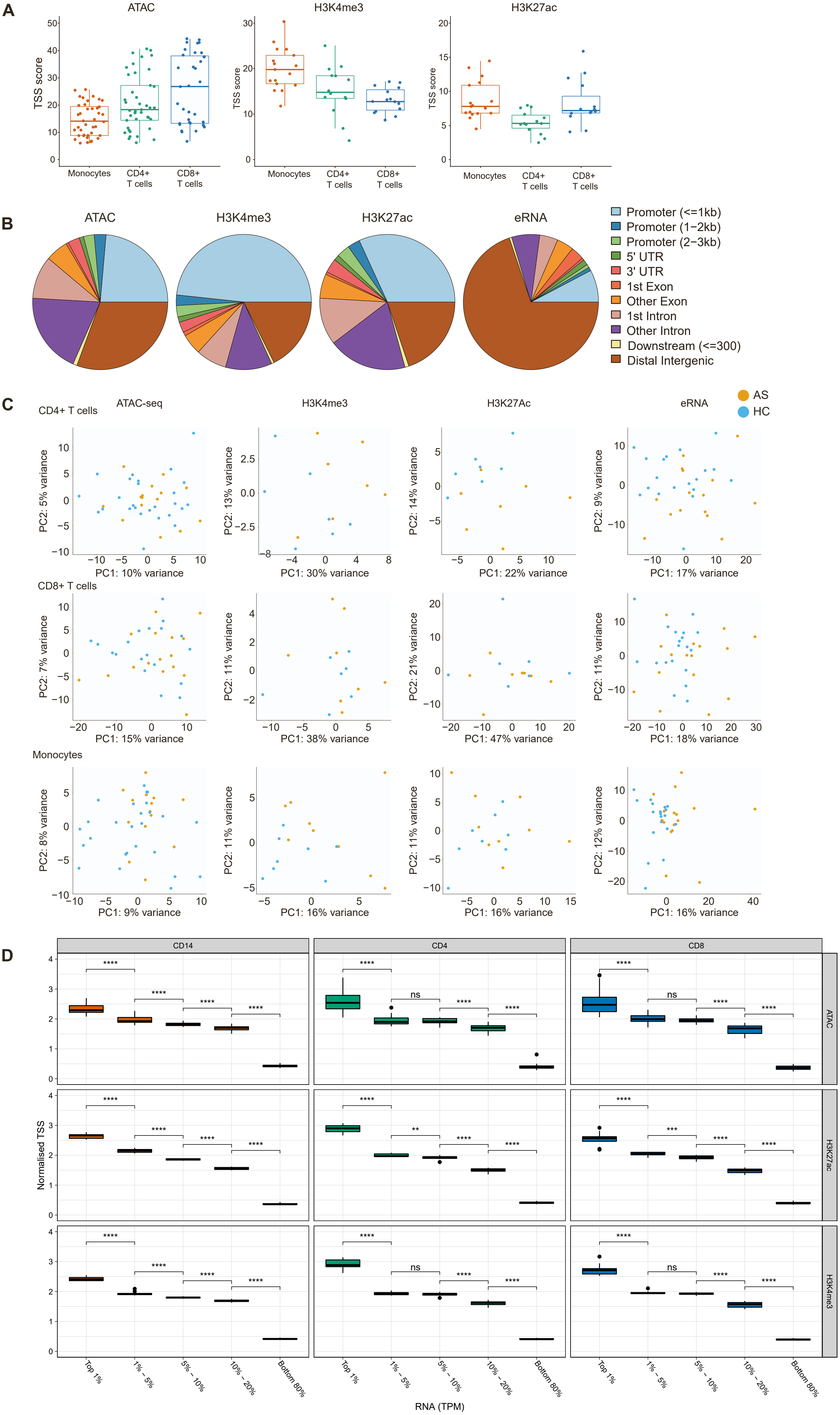
A
ATAC
H3K4me3
H3K27ac
TSS score
Monocytes
CD4+
T cells
CD8+
T cells
B
ATAC
H3K4me3
H3K27ac
eRNA
Promoter (<=1kb)
Promoter (1−2kb)
Promoter (2−3kb)
5' UTR
3' UTR
1st Exon
Other Exon
1st Intron
Other Intron
Downstream (<=300)
Distal Intergenic
C
CD4+ T cells
ATAC-seq
H3K4me3
H3K27Ac
eRNA
AS
HC
PC2: 5% variance
PC1: 10% variance
PC2: 13% variance
PC1: 30% variance
PC2: 14% variance
PC1: 22% variance
PC2: 9% variance
PC1: 17% variance
CD8+ T cells
PC2: 7% variance
PC1: 15% variance
PC2: 11% variance
PC1: 38% variance
PC2: 21% variance
PC1: 47% variance
PC2: 11% variance
PC1: 18% variance
Monocytes
PC2: 8% variance
PC1: 9% variance
PC2: 11% variance
PC1: 16% variance
PC2: 11% variance
PC1: 16% variance
PC2: 12% variance
PC1: 16% variance
D
CD14
CD4
CD8
ATAC
H3K27ac
H3K4me3
Normalised TSS
****
ns
**
***
Top 1%
1% − 5%
5% − 10%
10% − 20%
Bottom 80%
RNA (TPM)

Figure S2: Epigenomic marks are cell-type specific and have expected genomic distribution. Related to Figure 2.

(A) TSS enrichment scores for ATAC-seq and ChIPm (H3K4me3 and H3K27ac) samples in CD4+ T cells, CD8+ T cells and monocytes. Boxes represent the 25th and 75th percentile, the centre line represents the median. Upper and lower whiskers represent values with 1.5x the inter-quartile range.

(B) Genomic distribution of ATAC-seq, ChIP (H3K4me3 and H3K27ac) and eRNA peaks across all three cell types (generated with ChIPseeker).

(C) PCA showing distribution of ATAC-seq, ChIPm (H3K4me3 and H3K27ac) and eRNA peaks in AS patient and HC samples in each cell type (CD4+ T cells, CD8+ T cells and monocytes).

(D) TSS enrichment score of ATAC-seq and ChIPm (H3K4me3 and H3K27ac) peaks per ranked gene expression level of nearest gene. Boxes indicate the 25th and 75th percentile; whiskers indicate 1.5x the inter-quartile range. The centre line represents the median. P values calculated by Student's t-test between groups: $*p < 0.05$; $**p < 0.01$; $***p < 0.001$; $****p < 0.0001$.

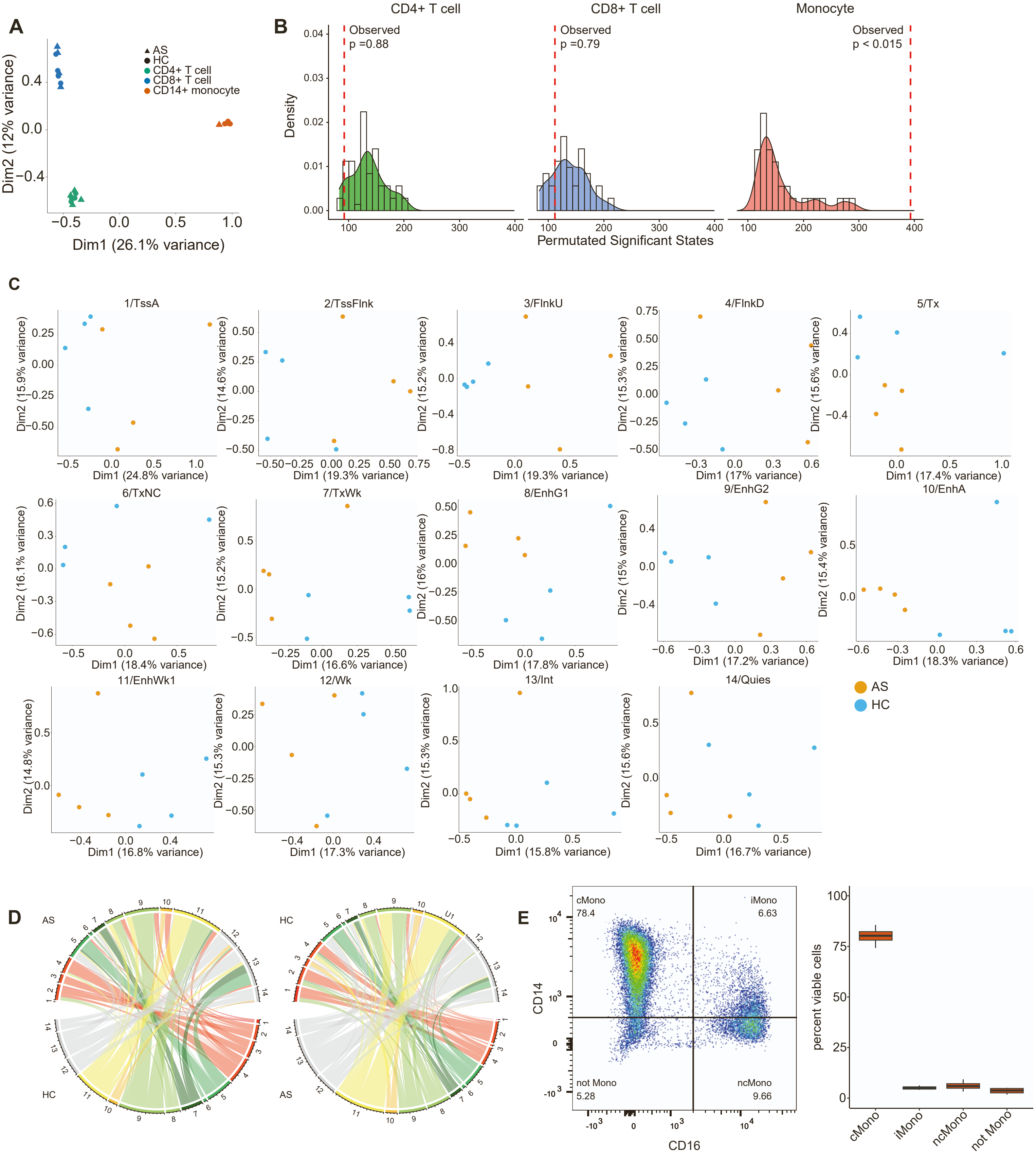

Figure S3: ChromHMM signatures are cell-type specific and differentiate AS patients and HC in monocytes. Related to Figure 3.

(A) MCA showing distribution of ChromHMM states across three cell types.

(B) Results of permutation analysis of AS-HC differences in ChromHMM state 10 for each cell type. The plot shows the number of significantly different states between AS and HC for each permutation (i.e. density). The experimentally observed frequency is shown with a red dashed line, and p value is indicated for each cell type. Significant p values are also indicated in Figure 3C.

(C) MCA showing distribution of individual ChromHMM states in monocytes, comparing AS patients and HC.

(D) Directionality of state transitions between equivalent genomic fragments in AS patients and HC in monocytes. Circle circumference represents the number of differential ChromHMM fragments (one tick = 200 fragments). Colours are consistent with Figure 3A. Left plot shows fragments present in 4 AS patients (upper half) and the state of that equivalent fragment in HC (bottom half). Right circle shows fragments present in 4 HC (upper half) and the state of the equivalent genomic fragment in AS patients (bottom half).

(E) Monocyte cell composition determined using flow cytometry. Representative plot from one individual (left panel); quantitation

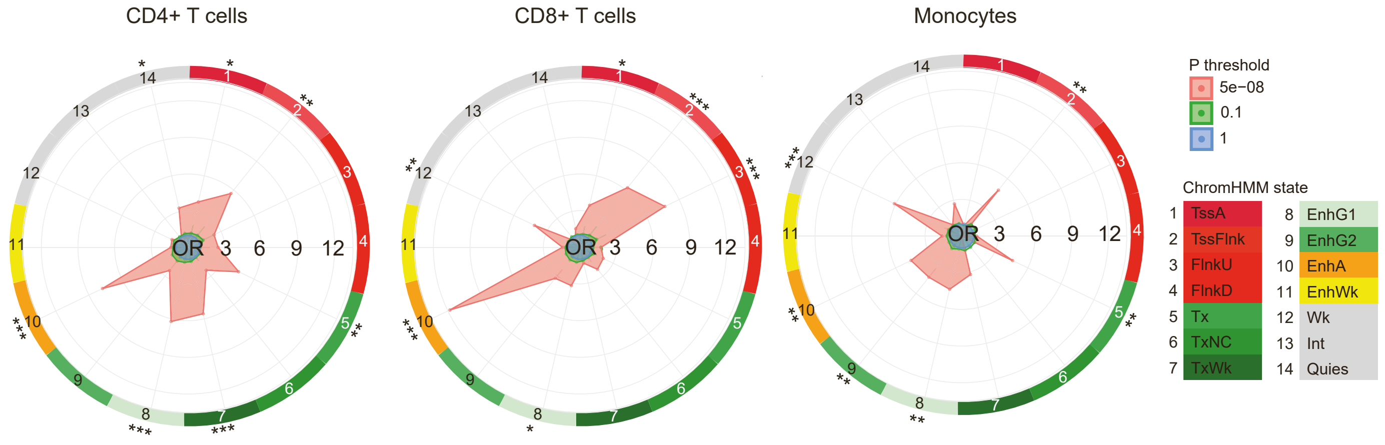

Figure S4: ChromHMM marks are enriched at AS-associated GWAS loci excluding the MHC in CD4+ T cells, CD8+ T cells and monocytes. Related to Figure 4.

Associated SNPs were included from Cortes et al9 with association p value thresholds as indicated. ChromHMM state from model in Figure 3. OR, Odds Ratio. ***p<0.001, **p<0.01, *p<0.05.

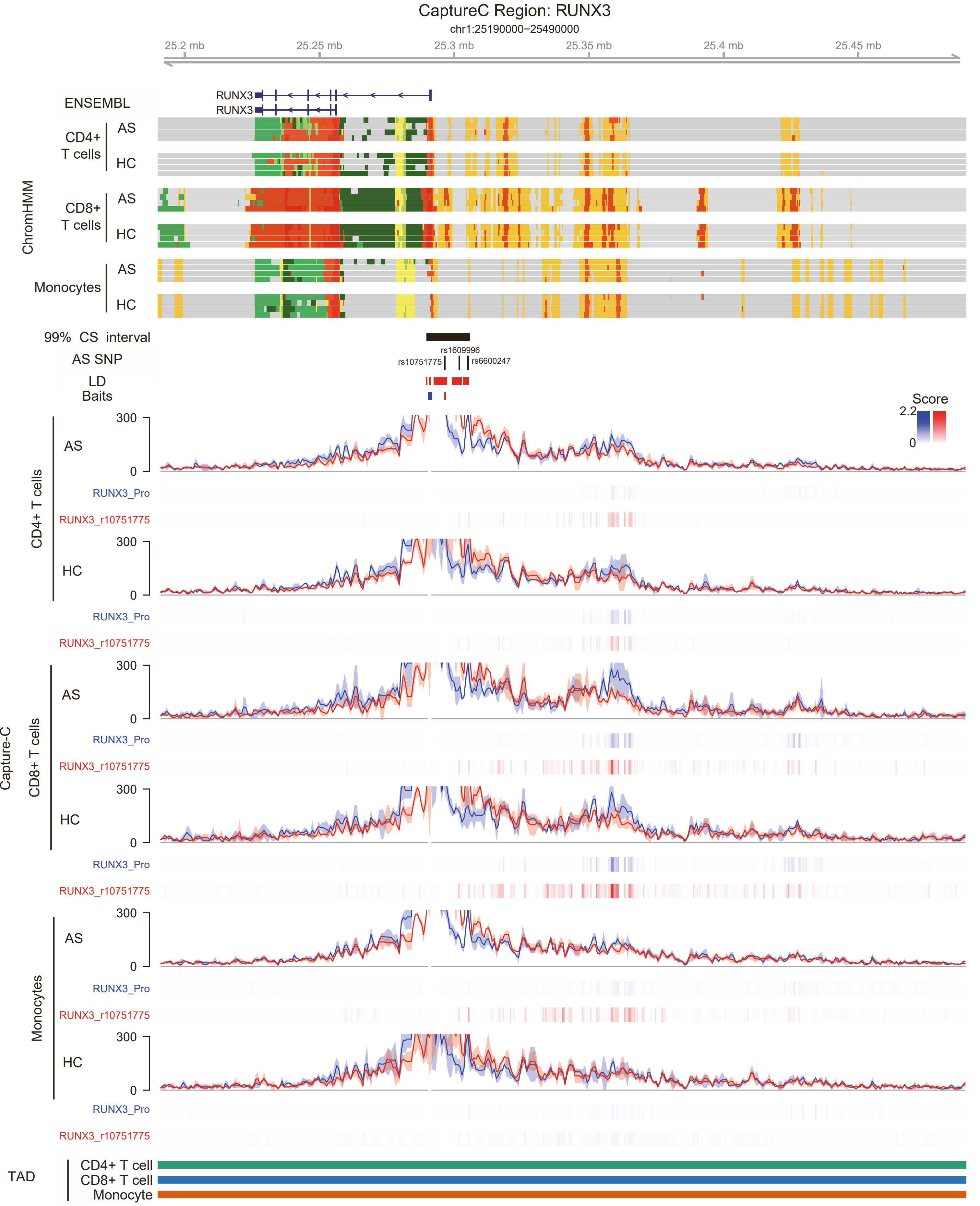

Figure S5: Enhancer-gene interactions at the *RUNX3* locus. Related to Figure 5 and Table 1.

Visualisation of interactions between AS-associated SNPs and the promoter of *RUNX3*.

Ensembl: selected transcripts of Ensembl genes; ChromHMM: data for each cell type from AS patients and HC are shown with colours as in Figure 3B; 99% CS interval: 99% credible set from van de Bunt et al[51]; AS SNP: Position of lead GWAS SNPs from Cortes et al[9] and/or Ellinghaus et al[10] as per Table 1; LD: SNPs in LD (r2 > 0.8) with lead SNP; Baits: Position of Capture-C baits (see also Table S8); Capture-C: Mean interaction count (n=3) and 1 sd shading, with PeakY scores coloured according to bait for AS patients and HC in CD4+ T cells, CD8+ T cells and monocytes; TAD: Topologically Associating Domain for each cell type.

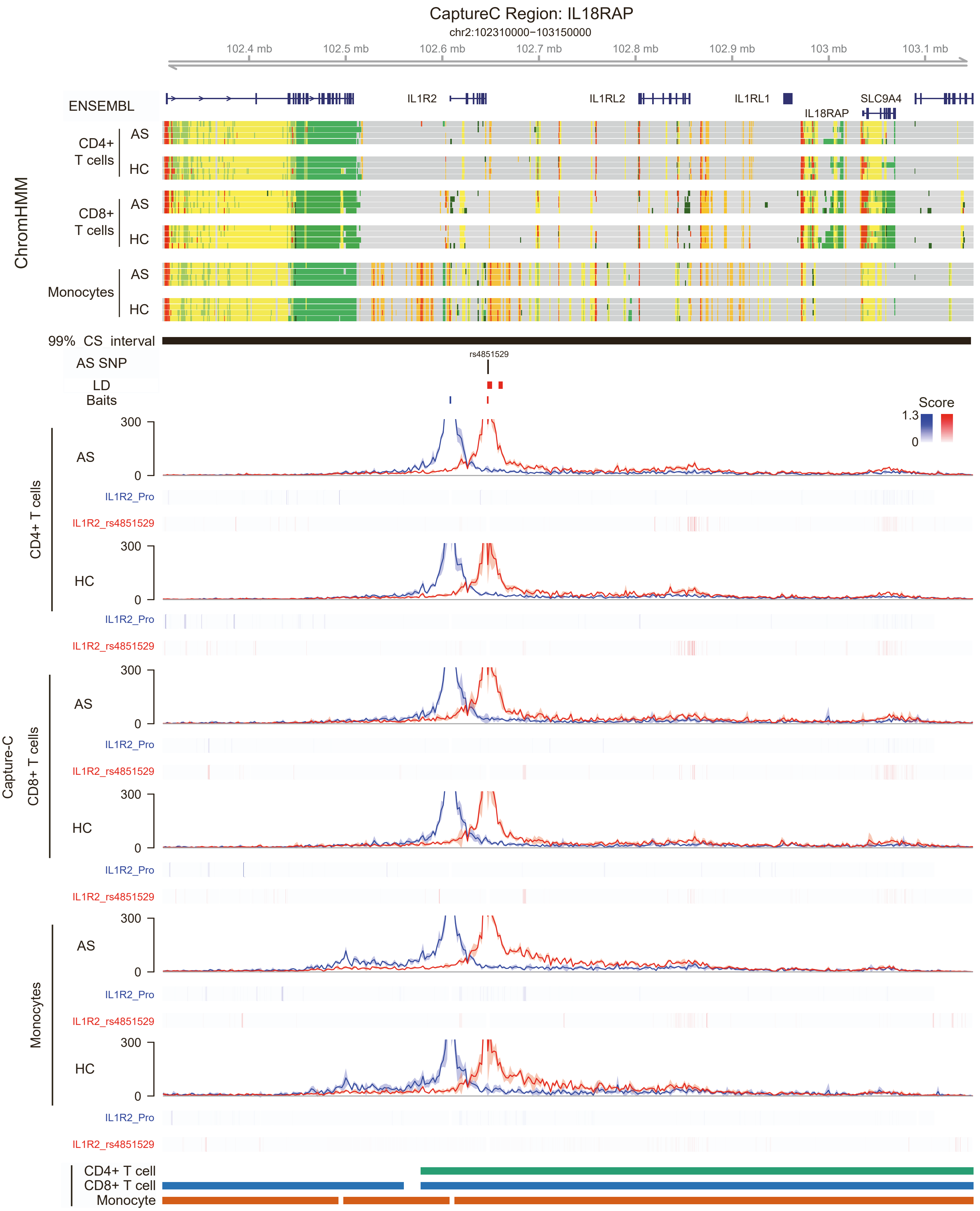

Figure S6: Enhancer-gene interactions at the *IL18RAP* locus. Related to Figure 5 and Table 1.

Visualisation of interactions between AS-associated SNPs and the promoter of *IL1RL2 and IL18RAP*.

Ensembl: selected transcripts of Ensembl genes; ChromHMM: data for each cell type from AS patients and HC are shown with colours as in Figure 3B; 99% CS interval: 99% credible set from van de Bunt et al[51]; AS SNP: Position of lead GWAS SNPs from Cortes et al[9] and/or Ellinghaus et al[10] as per Table 1; LD: SNPs in LD (r2 > 0.8) with lead SNP; Baits: Position of Capture-C baits (see also Table S8); Capture-C: Mean interaction count (n=3) and 1 sd shading, with PeakY scores coloured according to bait for AS patients and HC in CD4+ T cells, CD8+ T cells and monocytes; TAD: Topologically Associating Domain for each cell type.

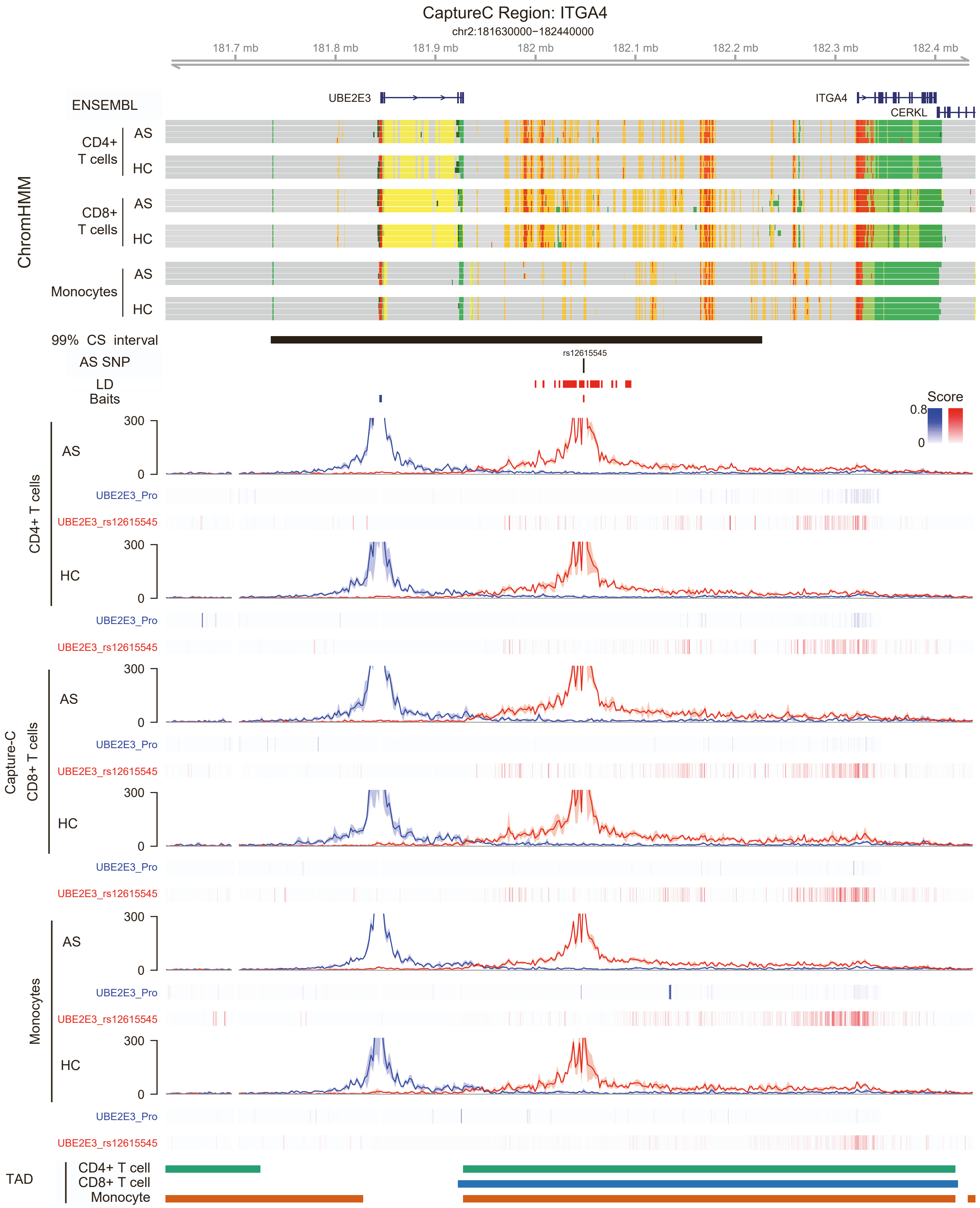

Figure S7: Enhancer-gene interactions at *the ITGA4* locus. Related to Figure 5 and Table 1.

Visualisation of interactions between AS-associated SNPs and the promoter of *ITGA4*.

Ensembl: selected transcripts of Ensembl genes; ChromHMM: data for each cell type from AS patients and HC are shown with colours as in Figure 3B; 99% CS interval: 99% credible set from van de Bunt et al[51]; AS SNP: Position of lead GWAS SNPs from Cortes et al[9] and/or Ellinghaus et al[10] as per Table 1; LD: SNPs in LD (r2 > 0.8) with lead SNP; Baits: Position of Capture-C baits (see also Table S8); Capture-C: Mean interaction count (n=3) and 1 sd shading, with PeakY scores coloured according to bait for AS patients and HC in CD4+ T cells, CD8+ T cells and monocytes; TAD: Topologically Associating Domain for each cell type.

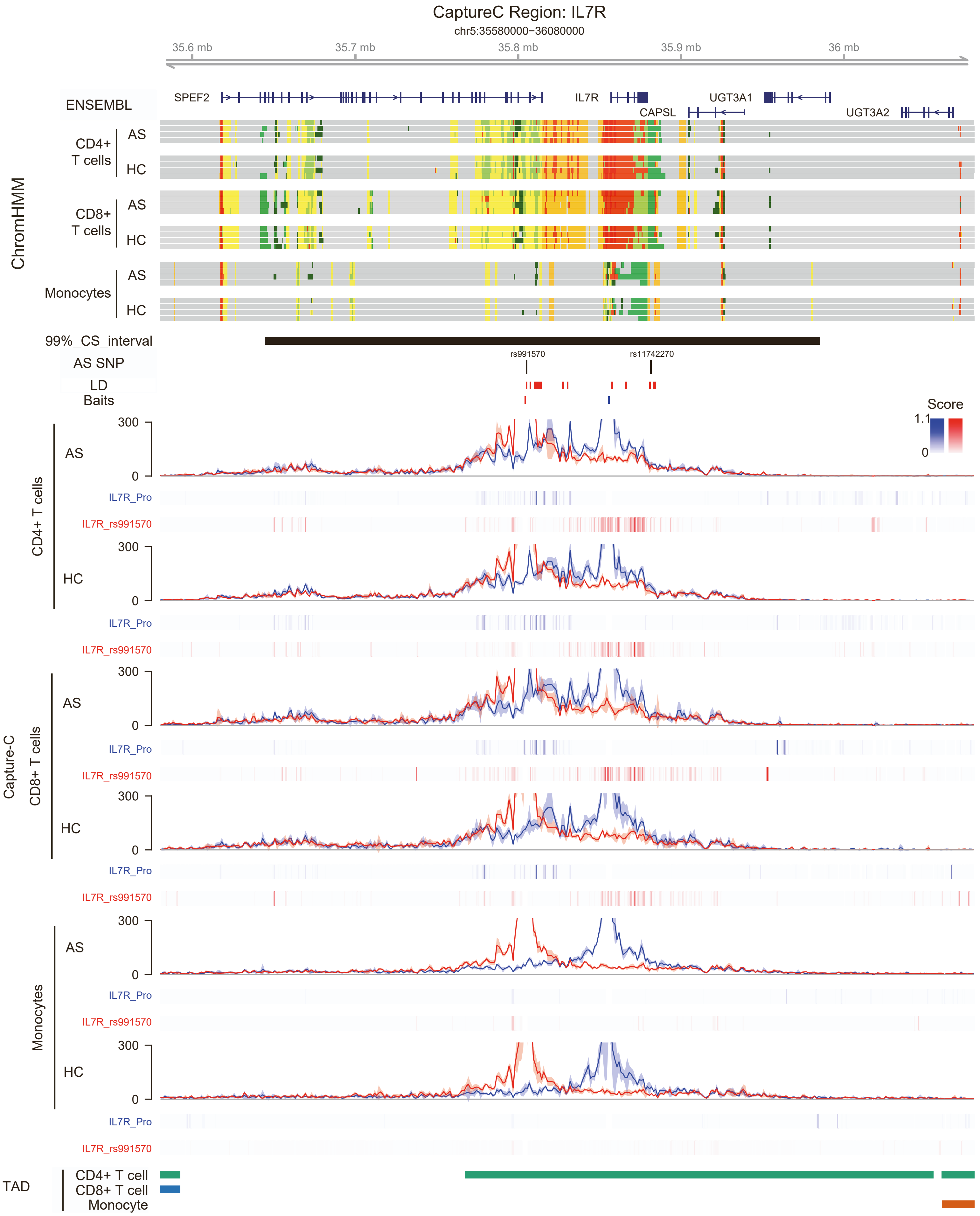

Figure S8: Enhancer-gene interactions at the *IL7R*locus. Related to Figure 5 and Table 1.

Visualisation of interactions between AS-associated SNPs and the promoter of *IL7R*.

Ensembl: selected transcripts of Ensembl genes; ChromHMM: data for each cell type from AS patients and HC are shown with colours as in Figure 3B; 99% CS interval: 99% credible set from van de Bunt et al[51]; AS SNP: Position of lead GWAS SNPs from Cortes et al[9] and/or Ellinghaus et al[10] as per Table 1; LD: SNPs in LD (r2 > 0.8) with lead SNP; Baits: Position of Capture-C baits (see also Table S8); Capture-C: Mean interaction count (n=3) and 1 sd shading, with PeakY scores coloured according to bait for AS patients and HC in CD4+ T cells, CD8+ T cells and monocytes; TAD: Topologically Associating Domain for each cell type.

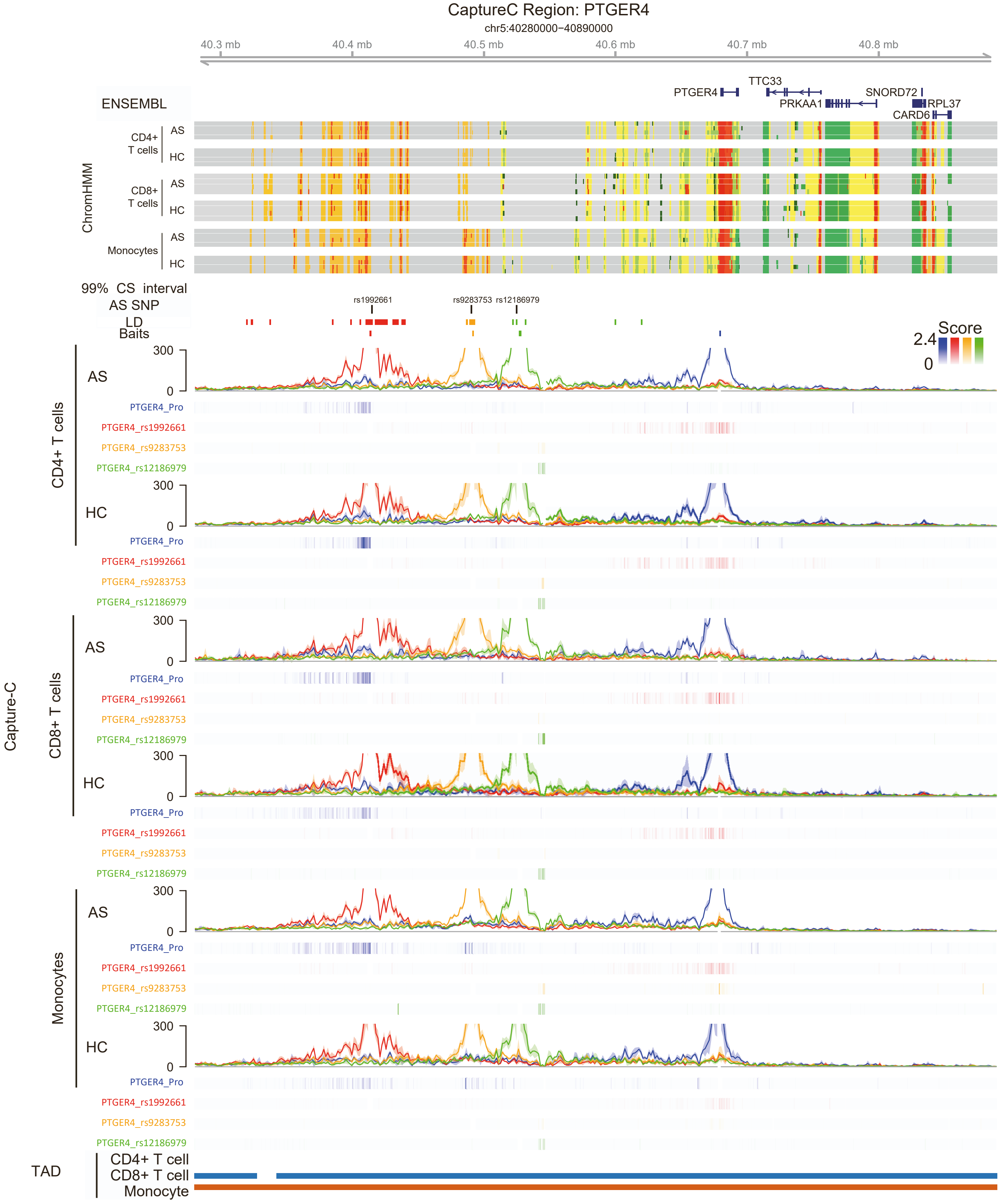

Figure S9: Enhancer-gene interactions at the *PTGER4*locus. Related to Figure 5 and Table 1.

Visualisation of interactions between AS-associated SNPs and the promoter of *PTGER4*.

Ensembl: selected transcripts of Ensembl genes; ChromHMM: data for each cell type from AS patients and HC are shown with colours as in Figure 3B; 99% CS interval: 99% credible set from van de Bunt et al[51]; AS SNP: Position of lead GWAS SNPs from Cortes et al[9] and/or Ellinghaus et al[10] as per Table 1; LD: SNPs in LD (r2 > 0.8) with lead SNP; Baits: Position of Capture-C baits (see also Table S8); Capture-C: Mean interaction count (n=3) and 1 sd shading, with PeakY scores coloured according to bait for AS patients and HC in CD4+ T cells, CD8+ T cells and monocytes; TAD: Topologically Associating Domain for each cell type.

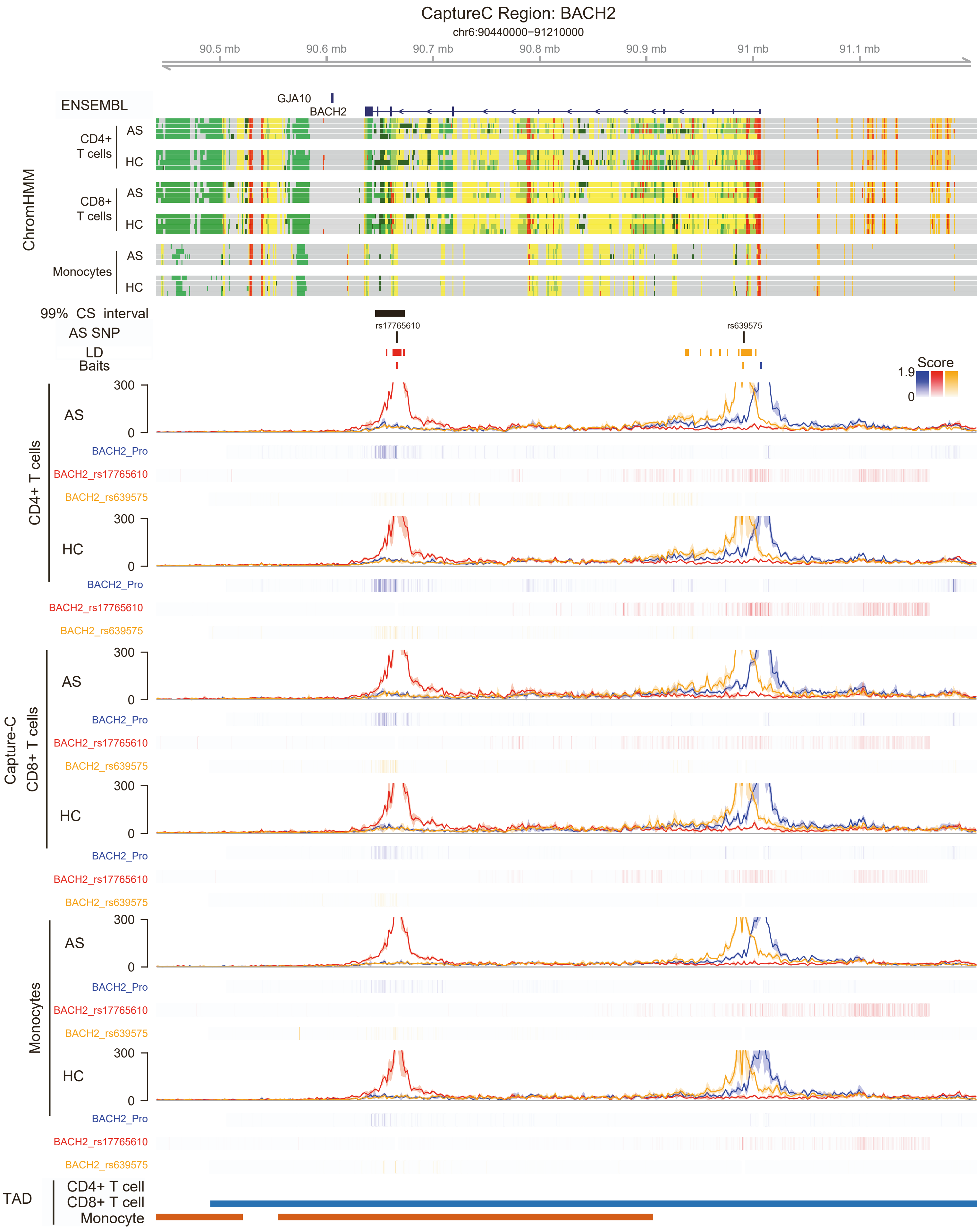

Figure S10: Enhancer-gene interactions at the *BACH2* locus. Related to Figure 5 and Table 1.

Visualisation of interactions between AS-associated SNPs and the promoter of *BACH2*.

Ensembl: selected transcripts of Ensembl genes; ChromHMM: data for each cell type from AS patients and HC are shown with colours as in Figure 3B; 99% CS interval: 99% credible set from van de Bunt et al[51]; Lead SNP: Position of lead GWAS SNPs from Cortes et al[9] and/or Ellinghaus et al[10] as per Table 1; LD: SNPs in LD (r2 > 0.8) with lead SNP; Baits: Position of Capture-C baits (see also Table S8); Capture-C: Mean interaction count (n=3) and 1 sd shading, with PeakY scores coloured according to bait for AS patients and HC in CD4+ T cells, CD8+ T cells and monocytes; TAD: Topologically Associating Domain for each cell type.

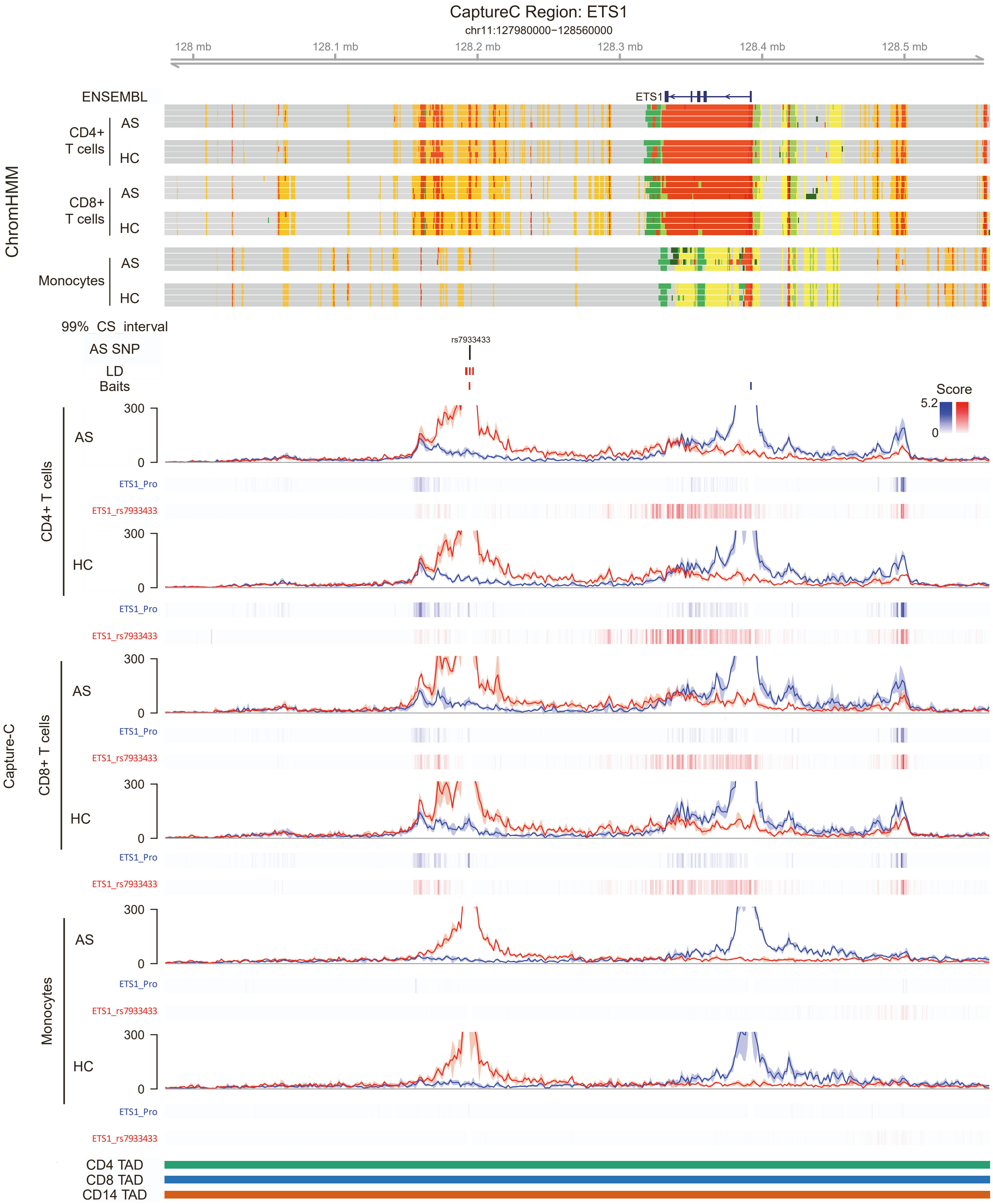

Figure S11: Enhancer-gene interactions at the *ETS1* locus. Related to Figure 5 and Table 1.

Visualisation of interactions between AS-associated SNPs and the promoter of *ETS1*.

Ensembl: selected transcripts of Ensembl genes; ChromHMM: data for each cell type from AS patients and HC are shown with colours as in Figure 3B; 99% CS interval: 99% credible set from van de Bunt et al[51]; AS SNP: Position of lead GWAS SNPs from Cortes et al[9] and/or Ellinghaus et al[10] as per Table 1; LD: SNPs in LD (r2 > 0.8) with lead SNP; Baits: Position of Capture-C baits (see also Table S8); Capture-C: Mean interaction count (n=3) and 1 sd shading, with PeakY scores coloured according to bait for AS patients and HC in CD4+ T cells, CD8+ T cells and monocytes; TAD: Topologically Associating Domain for each cell type.

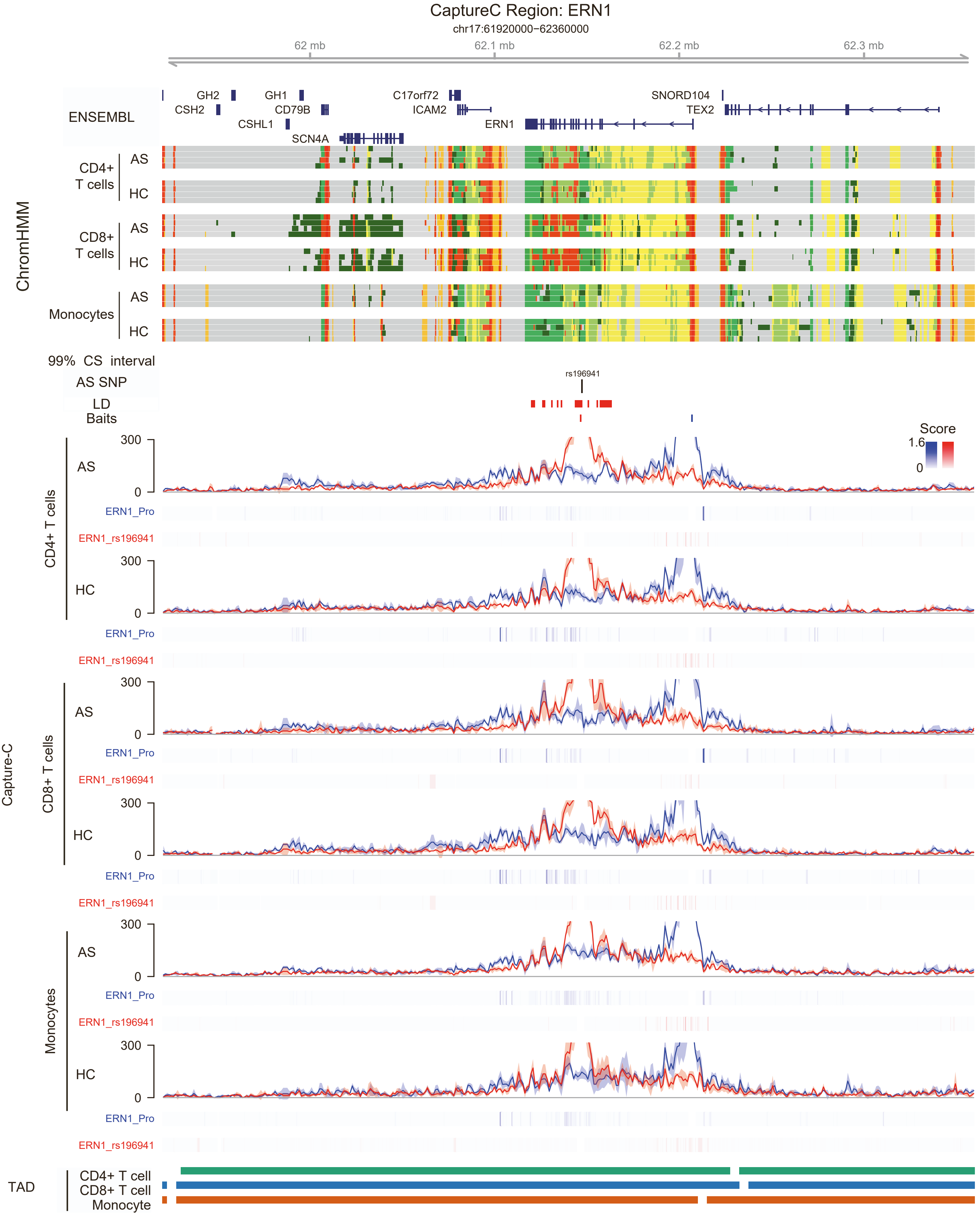

Figure S12: Enhancer-gene interactions at the *ERN1* locus. Related to Figure 5 and Table 1.

Visualisation of interactions between AS-associated SNPs and the promoter of *ERN1*.

Ensembl: selected transcripts of Ensembl genes; ChromHMM: data for each cell type from AS patients and HC are shown with colours as in Figure 3B; 99% CS interval: 99% credible set from van de Bunt et al[51]; AS SNP: Position of lead GWAS SNPs from Cortes et al[9] and/or Ellinghaus et al[10] as per Table 1; LD: SNPs in LD (r2 > 0.8) with lead SNP; Baits: Position of Capture-C baits (see also Table S8); Capture-C: Mean interaction count (n=3) and 1 sd shading, with PeakY scores coloured according to bait for AS patients and HC in CD4+ T cells, CD8+ T cells and monocytes; TAD: Topologically Associating Domain for each cell type.

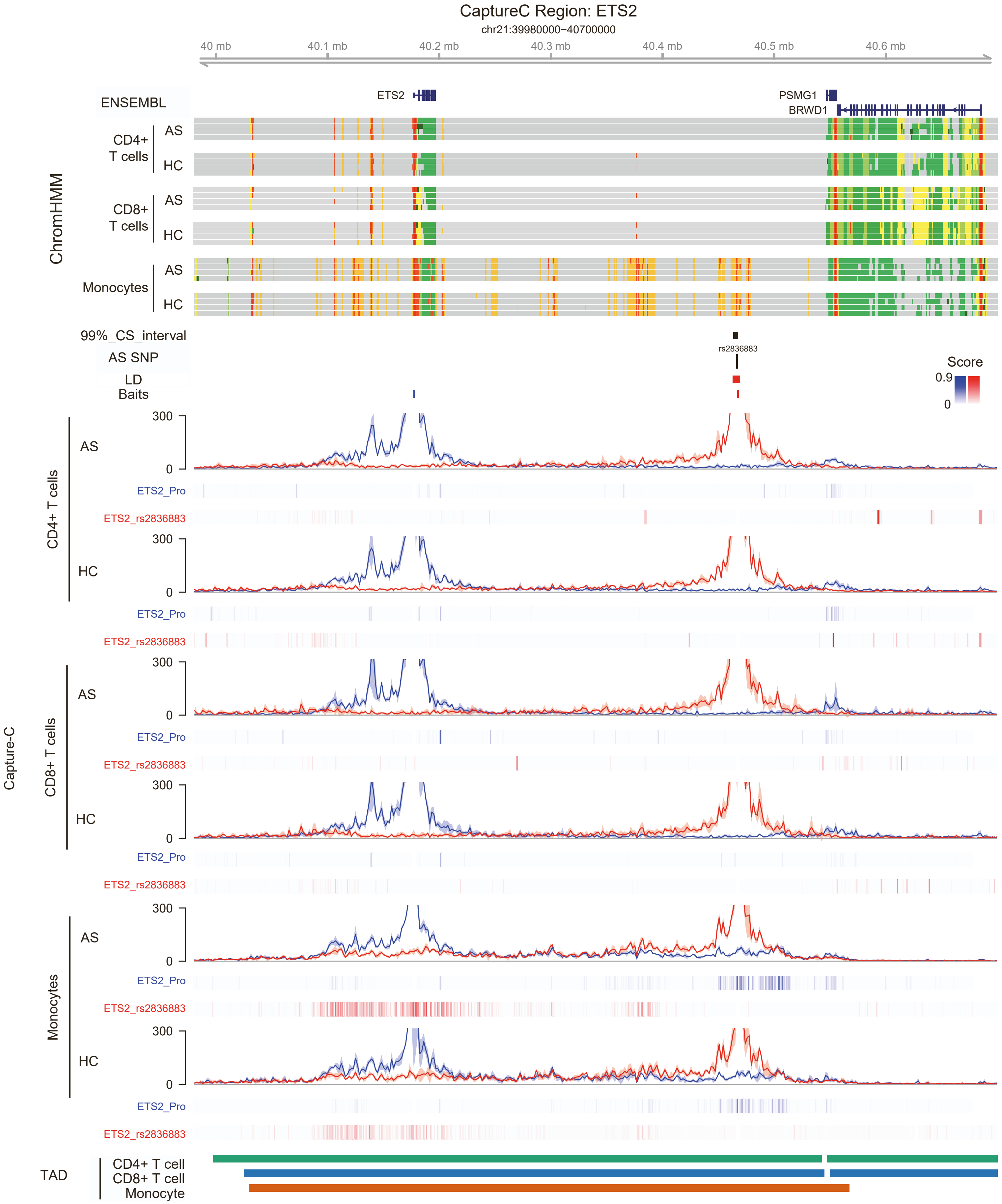

Figure S13: Enhancer-gene interactions at the *ETS2* locus. Related to Figure 5 and Table 1.

Visualisation of interactions between AS-associated SNPs and the promoter of *ETS2*.

Ensembl: selected transcripts of Ensembl genes; ChromHMM: data for each cell type from AS patients and HC are shown with colours as in Figure 3B; 99% CS interval: 99% credible set from van de Bunt et al[51]; AS SNP: Position of lead GWAS SNPs from Cortes et al[9] and/or Ellinghaus et al[10] as per Table 1; LD: SNPs in LD (r2 > 0.8) with lead SNP; Baits: Position of Capture-C baits (see also Table S8); Capture-C: Mean interaction count (n=3) and 1 sd shading, with PeakY scores coloured according to bait for AS patients and HC in CD4+ T cells, CD8+ T cells and monocytes; TAD: Topologically Associating Domain for each cell type.

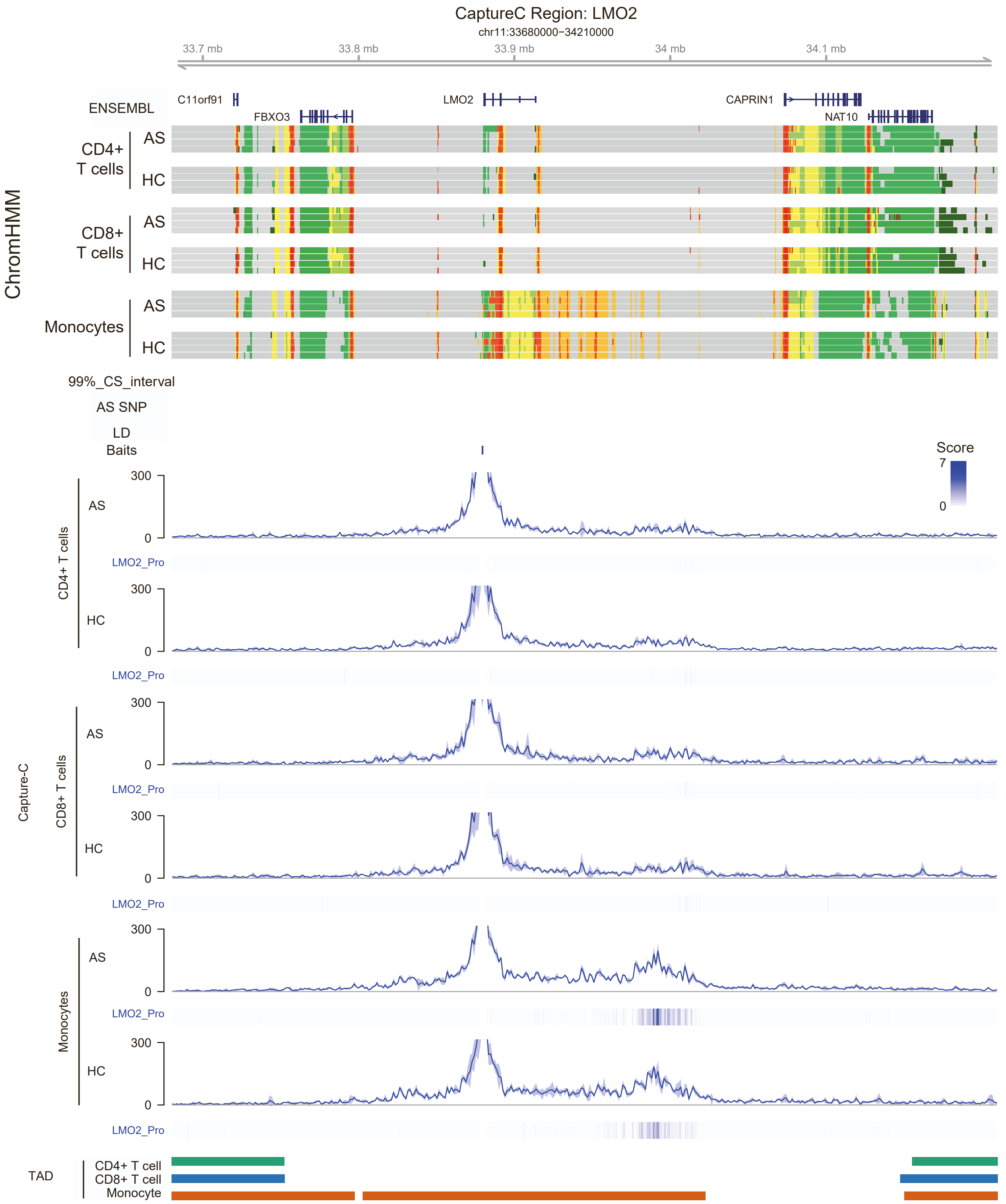

Figure S14: Enhancer-gene interactions at the *LMO2* control locus. Related to Figure 5 and Table 1.

Visualisation of interactions between a known enhancer and the promoter of *LMO2*.

Ensembl: selected transcripts of Ensembl genes; ChromHMM: data for each cell type from AS patients and HC are shown with colours as in Figure 3B; 99% CS interval: 99% credible set from van de Bunt et al[51]; AS SNP: Position of lead GWAS SNPs from Cortes et al[9] and/or Ellinghaus et al[10] as per Table 1; LD: SNPs in LD (r2 > 0.8) with lead SNP; Baits: Position of Capture-C baits (see also Table S8); Capture-C: Mean interaction count (n=3) and 1 sd shading, with PeakY scores coloured according to bait for AS patients and HC in CD4+ T cells, CD8+ T cells and monocytes; TAD: Topologically Associating Domain for each cell type.

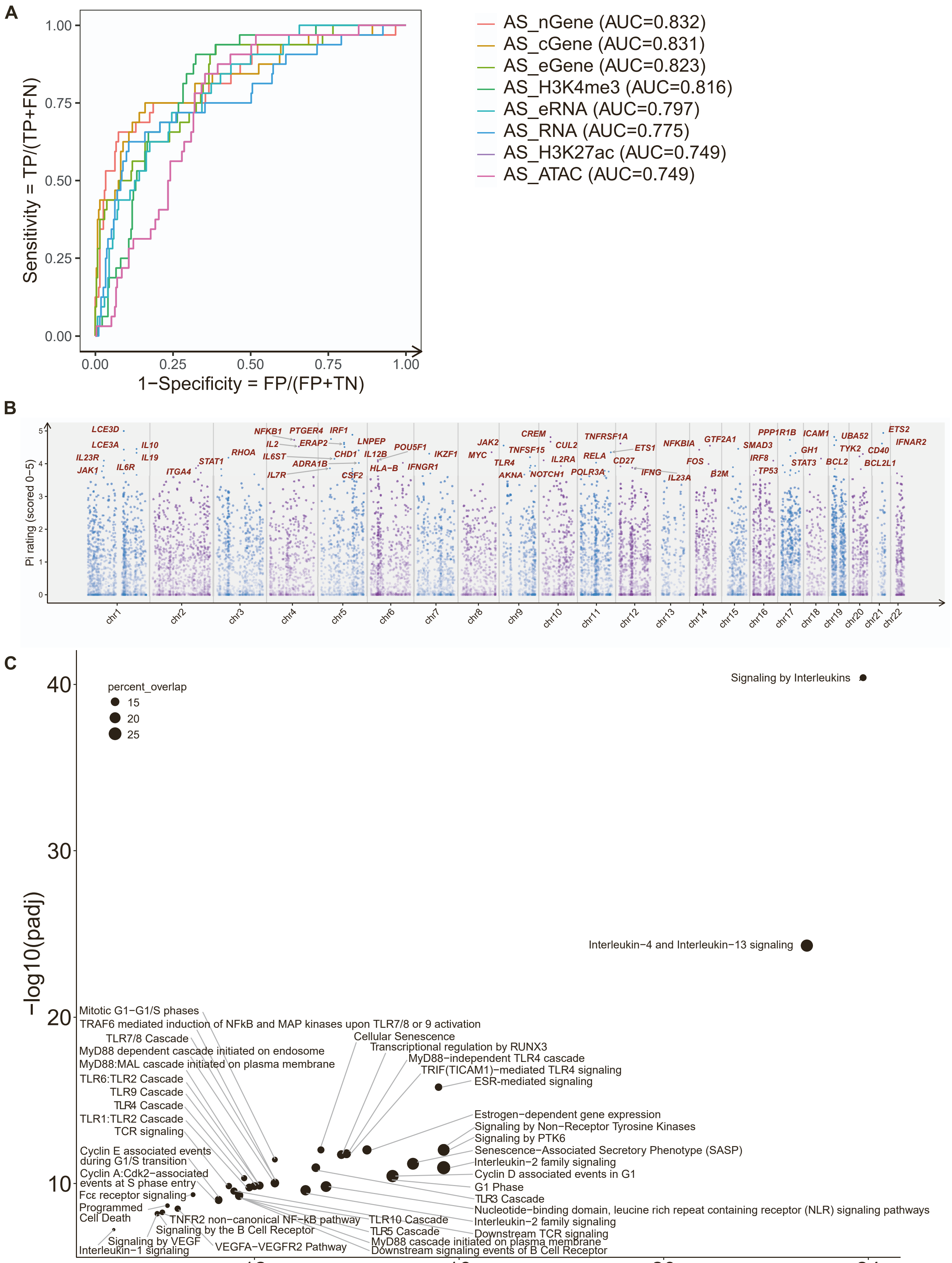

Figure S15: Incorporation of disease-specific data improves target discovery. Related to Figure 6.

(A) Performance of Pi algorithm on the basis of including each predictor type.

(B) Manhattan plot showing top 60 genes ranked by priority rating following inclusion of AS-specific data.

(C) Pathway analysis on the top 1% of Pi ranked genes using the Reactome database (padj < 0.05). Size of dots represents the percentage of overlapping genes in that pathway; only those with >10% overlap are shown.

Table S1: Clinical information for AS patients (AS) and HC. Related to Figure 1 and STAR Methods.

		AS	HC
Total number		20	35
Age (years)	Mean (range)	45 (28-74)	43 (21-67)
Sex (male)	N (%)	15 (75%)	17 (49%)
HLA-B27 (+)	N (%)	17 (85%)	5 (25%)[b]
Disease duration (months)	Mean (range)	128 (9-456)	N/A
BASDAI (units NRS)	Mean +/- SD	5.9 +/- 1.7	N/A
ASDAS-CRP	Mean +/- SD	3.2 +/- 1.1	N/A
CRP, mg/L	Mean +/- SD	13.5 +/- 22.8	N/A
Axial manifestation of SpA	N (%)	20 (100%)	0 (0%)
Peripheral articular manifestations			
• Peripheral arthritis	N (%)	8 (40%)	0 (0%)
Extra-articular manifestations			
• Psoriasis	N (%)	3 (15%)	0 (0%)
• Uveitis	N (%)	5 (25%)	0 (0%)
• Inflammatory Bowel Disease	N (%)	1 (5%)	0 (0%)
Other comorbidities[a]			
• Cancer	N (%)	1 (5%)	0 (0%)
• Fracture	N (%)	6 (30%)	0 (0%)
• Vertebral fracture	N (%)	1 (5%)	0 (0%)
• Autoimmune Thyroid Disease	N (%)	1 (5%)	0 (0%)
Drugs (current)			
• NSAIDs	N (%)	12 (60%)	0 (0%)
• csDMARDs	N (%)	3 (15%)	0 (0%)
• Glucocorticoids	N (%)	0 (0%)	0 (0%)
Family history			
• AS	N (%)	7 (35%)	Unknown
• Other autoimmune disease	N (%)	7 (35%)	Unknown

[a] No incidences of aortic disease, atlantoaxial subluxation, coeliac disease, dermatomyositis, fibrotic lung disease, haemolytic anaemia, heart block, multiple sclerosis, myasthenia gravis, pemphigus vulgaris, pernicious anaemia, scleroderma, systemic lupus erythematosus, type 1 diabetes, vitiligo.

[b] Not all HC have imputed B27, percentage is calculated on 20 HC that have genotyping.

BASDAI, Bath Ankylosing Spondylitis Disease Activity Index; NRS, Numerical Rating Scale; ASDAS, Ankylosing Spondylitis Disease Activity Score; CRP, C-Reactive Protein; SpA,

Spondyloarthropathy; NSDAIDs, Non-Steroidal Anti-Inflammatory Drugs; csDMARDs, Conventional Synthetic Disease-Modifying Antirheumatic Drugs.

Supplemental References

S1. Cortes A., Hadler J., Cremin K., Pryce K., Harris J., Pointon J.P., Evans D.M., Leo P., Robinson P., Bradbury L.A., et al. (2013). Identification of multiple risk variants for ankylosing spondylitis through high-density genotyping of immune-related loci. Nat Genet. 45, 730–738.

S2. van de Bunt M., Cortes A., Consortium I., Brown M.A., Morris A.P., McCarthy M.I. (2015). Evaluating the Performance of Fine-Mapping Strategies at Common Variant GWAS Loci. PLoS Genet. 11, e1005535. 10.1371/journal.pgen.1005535

S3. Ellinghaus D., Jostins L., Spain S.L., Cortes A., Bethune J., Han B., Park Y.R., Raychaudhuri S., Pouget J.G., Hübenthal M., et al. (2016). Analysis of five chronic inflammatory diseases identifies 27 new associations and highlights disease-specific patterns at shared loci. Nat Genet. 48, 510. 10.1038/ng.3528
